# Inter-provincial embodied carbon emission space and industrial transfer paths in China

**DOI:** 10.1371/journal.pone.0300478

**Published:** 2024-06-27

**Authors:** Wenying Zhang, Mengqi Yang, Jianhua Ge, Gangzhen Wang

**Affiliations:** 1 Business School, China University of Political Science and Law, Beijing, China; 2 School of Finance, Anhui University of Finance and Economics, Bengbu, China; Hong Kong Shue Yan University, HONG KONG

## Abstract

To bolster the dual-circulation development model and green economy, this study delves into the spatiotemporal dynamics of implied carbon transfer in China’s inter-provincial and inter-industrial trade, emphasizing its significance for the "dual carbon" objectives. Utilizing multi-regional input-output data from 2012, 2015, and 2017, we employed the multi-region input-output model to gauge embodied carbon transfers across 31 provinces and 28 industries. The Structural Decomposition Analysis (SDA) model further decomposed the growth of trade-related carbon emissions. Key findings include: (1) The electricity and thermal power sectors dominate carbon transfers, with secondary industries seeing rapid growth; (2) Northern provinces significantly outweigh southern ones in carbon transfers and the main direction of it is towards affluent southern coastal regions; (3) Scale effect plays a pivotal role in these transfers. Conclusively, it is crucial for regulatory authorities to rationally formulate region-specific emission policies for inter-regional coordination, and future studies can focus on industrial and spatial clustering effects.

## 1 Introduction

As global industrialization accelerates, the demand for energy is increasing correspondingly. Although energy drives economic growth, it also increases carbon emissions, especially in small-scale economies where increased energy consumption leads to increased carbon emissions [1]. The concept of "embodied carbon" includes both direct and indirect resource consumption during the whole production process (from raw material acquisition and final product output) [2]. This concept is put forward to emphasize the part of carbon emissions hidden in materials and manufacturing processes, as well as in imports and exports activities between nations [3]. With the increase of industrial demand and trade division between countries and regions, industries or products with high carbon emissions (carbon sources) flows between regions, causing the transfer of embodied carbon’s emissions, which is called carbon transfer [4]. China is a major exporter of embodied carbon, and there are regional variations in resource allocation and economic growth. China needs to measure the inter-regional embodied carbon emission transfers accurately to localized emission reduction strategies.

Recent research had predominantly used the 2017 dataset of China’s Multi-regional input-output. For instance, Xing et al.’s [5] research narrowed down the scope to 29 cities in the Central Plains Urban Agglomeration. It explored city-level carbon footprints and interregional CO2 transfers in the trade of finished products across these cities [5]. Li et al. [6] further focused on Shanxi Province, an energy-dense Chinese region and conducted a more nichetargeting research. Compared with them, our key contributions include: (1) Different from previous literatures which merely focus on some point in time, our study analyses the embodied carbon transfers in 2012, 2015, and 2017, providing more details about industry-specific dynamics. (2) While most researches regarding carbon emissions from China’s foreign trade focus on the factors influencing embodied carbon emissions, few venture into industry-specific and regional-specific explorations, particularly the disparity in embodied carbon emissions efficiency across different industries and provinces. This study takes 28 sub-industries as research objects, and meticulously examines the embodied carbon transfer in different industrial patterns in China. (3) Different from conventional research, our study provides a more detailed investigation through exploring the “embodied” carbon transfers between China’s 30 provinces, and pinpoints the determinants of trade’s embodied carbon transfers.

The aims of the paper are as follows: (i) to study the growth patterns spanning industrial sectors and inter-provincial domains as well as the structural shifts in China’s embodied carbon transfers; (ii) to study the trajectories and overarching characteristics of China’s inter-provincial embodied carbon emissions; (iii) to study the determinants of trade’s embodied carbon transfers.

## 2 Literature review

The literature on embodied carbon emission transfer in China can be broadly divided into two categories [7, 8]. The first encompasses Multi-Regional Input-Output-centred analyses of China’s inter-regional embodied carbon flows. The other literature generally involved SDA (Structural Decomposition Analysis)—a detailed analysis of the complexity of regional emissions in China.

### 2.1 Multi-regional input-output analysis of China’s regional embodied flows

Recent research had predominantly used the 2017 dataset of China’s Multi-regional input-output. Multi-Regional Input-Output (MRIO) model is used to compute the net transfer of embodied carbon emissions, and it provides a comprehensive view of input-output relationships across regions and industries, capturing both direct and indirect impacts of trade dynamics. It encapsulates the ripple effects of trade across countries and regions and covers all the indirect consequences of previous production activities. S1 Table showed summary of literature on MRIO analysis of China’s regional embodied flows. Wang et al. [9] innovatively uses the multi-regional Input-Output to analyse the concrete embodiment of carbon emissions in bilateral trade. They integrated carbon emission intensity with the input-output model to gauge interregional carbon transfers within China’s national value chain from the perspectives of forward linkage, backward linkage, and trade in value-added, providing a unique perspective for studying the impact of international trade on the environment [9]. Dong et al. [10] used the multi-regional input-output model to explore the dynamic shifts in China’s industrial carbon footprint from 2012 to 2017. They first estimated the carbon emissions of different sectors in China based on energy consumption data and emission coefficients [10]. Then they delved into production and consumption carbon transfers, inter-provincial net carbon transfers, and export net carbon transfers using the multi-regional input-output table and China’s carbon data. Nevertheless, the study relies on extensive data sources and modelling, which may limit its applicability in regions or countries with less robust data infrastructure.

Xing et al.’s [5] research narrowed down the scope to 29 cities in the Central Plains Urban Agglomeration. It explored city-level carbon footprints and interregional CO2 transfers in the trade of finished products across these cities [5]. By constructing a multi-regional nested input-output table, they found that emissions due to capital formation were paramount in contributing to the carbon footprint, followed closely by urban consumption [11, 12]. Li et al. [6] further focused on Shanxi Province, an energy-dense Chinese region and conducted a more nichetargeting research. It combined the multi-regional input-output model with the structural decomposition analysis approach to investigate the spatiotemporal dynamics and drivers of embodied carbon emissions. They found that the significant embodied carbon outflows from Shanxi are driven primarily by technological advances and trade demands [6], however, broader factors, such as policy changes and economic development patterns, which may also influence these transfers, are not extensively explored. In addition, they also found that the major industries with net embodied carbon outflows encompassed electricity and heat production, metal processing, and oil processing.

Considering the diverse circumstances at home and abroad, Justin et al. [13], looking beyond China, used the multi-regional input-output framework to gauge both direct and indirect CO2 consumption across US regions. They enriched existing research by considering emissions from both domestic and international imports, drawing from detailed bilateral trade data between US states and global regions [13]. The multi-regional input-output model elucidated novel characteristics of embodied carbon emissions, while the structural decomposition analysis shed light on the primary drivers of the shifts in embodied carbon emission patterns, revealing a pronounced north-to-south emission transfer trend in 2017.

### 2.2 Structural decomposition analysis of China’s regional emission issues

S2 Table showed the overview of Literature on SDA of China’s Regional Emission Issues. A series of studies have been conducted on the regional environmental and economic challenges in China using the Structural Decomposition Analysis (SDA) method. Su and Ang [14] provided an empirical comparison of four distinct structural decomposition analysis techniques using the CO2 emission data of China and the study investigated the similarities and differences between SDA and IDA and found that the gap between SDA and IDA with regard to the decomposition methods used has not widened but instead narrowed [14]. Their work offered critical guidance on selecting the most appropriate methodological approach.

Most scholars employed SDA method to conduct research related to China’s energy patterns, especially focus on the drivers of energy consumption and carbon emissions. In a comprehensive energy review across 31 Chinese regions, Yan et al. [15] discovered that, due to investment, consumption, and exports, the eastern region predominantly shapes China’s energy patterns, having superior embodied energy consumption and reduced intensity [15]. With a combined approach of input–output analysis (IOA) and biproportional scaling method (RAS), Peng et al. [16] found that income-based household emissions exceeded consumption-based ones from 2010 to 2017 [16]. Nevertheless, the study focuses on the environmental aspects, but it doesn’t discuss the broader socio-economic implications of its findings, especially considering China’s rapid urbanization and changing economic landscape.

Other researches paid attention to the drivers of energy consumption and corresponding carbon emissions. For instance, Wang et al. [17] used structural decomposition analysis to investigate in depth the driving forces behind energy consumption and related emission fluctuations in China [17]. Their findings are conducive for administering authorities to evaluate and formulate effective energy and climate policies. Different from Wang et al [17], Tang et al. [18] Innovatively introduced a layered structural decomposition analysis method, revealing the effects of production substitution on China’s embodied carbon emissions from 1997 to 2017 [18].

Some studies narrowed the research scope to a specific region. Cheng et al. [19] pinpointed the carbon footprint and its socio-economic drivers in the Yangtze River Delta’s urban agglomerations [19]. Highlighting Jiangsu Province’s CO2 emissions, Xu et al. [20] attributed the rise in emissions to provincial economic growth, especially transfer and investment effects [20].

Liu et al. [21] and Huang et al. [22] further extended the use of structural decomposition analysis, examining urban household carbon emissions and provincial energy changes respectively, during periods of rapid economic development [23, 24]. They also explored the inter-regional spillover effects. Collectively, these studies demonstrate the ability and precision of the structural decomposition analysis (SDA) approach in assessing regional emission issues in China. However, given the focus on spatial and regional heterogeneity, there might be an overemphasis on regional patterns, potentially neglecting macro factors.

Our research stands out for several pivotal reasons. While most studies emphasize embodied carbon emissions from international trade, this paper uniquely addresses China’s embodied carbon transfers within its industrial sectors and provinces. In 1979, Walter and Ugelow introduced the concept of the "pollution haven hypothesis" [25]. This primarily alludes to the scenario where countries with stringent environmental regulations offload high-pollution industries to nations with more lenient environmental policies, aiming to meet their own strict emission standards. Given China’s drive towards a green economy and the dual-circulation development model, understanding these domestic transfers becomes important for formulating suitable carbon reduction policies. Using the Multi-Regional Input-Output model, the study calculates net carbon transfers for 2012, 2015, and 2017, offering a holistic view by integrating industry linkage effects with other decomposition techniques. By transitioning from single-year data to a multi-year panel format, the research presents a temporal exploration of embodied carbon transfers both at the industry level and between provinces. We aim to discern the pathways, growth rates, and overarching patterns of China’s interprovincial embodied carbon emissions, thus facilitating the development of region-specific and industry-tailored carbon reduction strategies.

## 3 Theoretical framework and hypotheses development

### 3.1 Embodied carbon transfer between industrial sectors in China

In an open economic framework, trade in goods and services inherently carries with it an exchange of embodied resources such as capital, energy, technology, and labour. The concept of "embodied flows" encompasses the virtual transfer of materials wherein tangible components are either directly or indirectly consumed in the production process, making them inherent to the resultant product or service. By leveraging the "embodied flow" theory, we can gain a profound understanding of the origins and trajectories of embodied resources in trade activities [26]. This perspective elucidates the genuine benefits and environmental costs borne by the trading entities. In the current epoch marked by rising sea levels and extreme climatic events, induced by global climate change, there is a heightened focus on greenhouse gas emissions and the transfer of embodied carbon within industrial sectors. China is the owner of the world’s largest power system, and the carbon emissions from China’s power sector accounts for about 40% of the country’s total energy-related emissions [27]. As such, it’s imperative to recognize and address the carbon footprint arising from the substitution of electricity in various industries and the electrification of end-use energy consumption. This focus not only serves as a pivotal strategy for carbon reduction but is also central to the transition towards cleaner, low-carbon energy consumption. Endowed with vast coal reserves, China’s power and heating sectors predominantly rely on coal, complemented by hydropower and wind energy. Given this reliance, carbon dioxide emissions from China’s power sector rank the highest, underscoring the sector’s pivotal role in achieving the nation’s "dual carbon" objectives. It refers to “carbon peaking” and “carbon neutrality”. Carbon peaking means that the annual carbon dioxide emissions of a certain region or industry reach an all-time high, and then enter the process of continuous decline through the plateau period, which is the historical inflection point of carbon dioxide emissions from growth to decline, marking the decoupling of carbon emissions and economic development; Carbon neutrality refers to the total amount of greenhouse gas emissions directly or indirectly generated by enterprises, groups or individuals in a certain period of time, and they offset their own carbon dioxide emissions through afforestation, energy conservation and emission reduction, etc., to achieve carbon dioxide "zero emissions". The 2020 Central Economic Work Conference made it clear that China’s carbon dioxide emissions will strive to peak before 2030 and strive to achieve carbon neutrality before 2060. Concurrently, the advanced manufacturing sector in China is primarily powered by electricity and heat. The embodied CO2 levels in their consumption mirror the nation’s industrial development trajectory.

The secondary industry, predominantly characterized by its industrial nature, is marked by substantial resource consumption due to its vast scale of operations. Historically, the growth of traditional industries has acted as a major catalyst for expansive economic development, consequently leading to a surge in energy demands. As the secondary industry evolves, there is a notable uptick in carbon emissions [28]. In contrast to the tertiary sector, the secondary sector exhibits a higher resource consumption rate, which in turn generates a greater volume of pollutants and carbon emissions.

Based on the above analysis, the following hypotheses are proposed:

H 1a: Regarding the embodied carbon transfers among China’s industrial sectors, the electric power and heat industry accounts for the largest proportion.
H 1b: Regarding China’s inter-sectoral embodied carbon transfer, the secondary industry has the highest embodied carbon growth rate.

### 3.2 Inter-provincial embodied carbon transfer in China

Global warming looms as an urgent challenge to humanity, with its escalating effects permeating every facet of life. Over the years, the input-output theory has been instrumental for scholars delving into the ramifications of economic and trade activities on the environment, primarily examining carbon emission flows induced by trade. The renowned American economist, Leontief, introduced the industry correlation concept, or the "input-output" theory, in 1928 [29]. The input-output table he formulated elucidates the intricate interplay between each industry’s intermediate input and output, offering a lucid depiction of an industry’s stance within an economic system. Leveraging the input-output model, the multi-regional input-output table serves as a tool to gauge carbon emissions across various sectors and their interconnections within specific regions. Furthermore, a multi-regional input-output table aids in assessing the environmental repercussions of inter-provincial trade. Building on the foundation of the input-output theory, researchers can ascertain carbon emissions and their ensuing transfers attributable to trade, production, and consumption across individual regions. This study contributes a theoretical framework for dissecting the transfer of embodied carbon emissions across China’s diverse regions.

In 1979, Walter and Ugelow introduced the concept of the "pollution haven hypothesis" [25]. This primarily alludes to the scenario where countries with stringent environmental regulations offload high-pollution industries to nations with more lenient environmental policies, aiming to meet their own strict emission standards. Such a trend is predominantly observed in many developed countries where rapid economic progress, coupled with heightened environmental consciousness among the populace, necessitates higher ecological standards. Consequently, these nations strategically relocate their high-emission, polluting industrial sectors to developing countries.

In line with the pollution haven hypothesis, many cities in northern China are characterized by a dominance of heavy industries, a prevalence of resource-based cities, and a lag in the evolution of innovative growth drivers, leading to constrained green development. These factors, coupled with stark developmental disparities between the North and South, result in pronounced environmental degradation, elevated carbon emissions, and substantial embodied carbon in trade for other regions. Furthermore, northern provinces in China exhibit greater embodied carbon transfers than their southern counterparts. Concurrently, due to the varied pace of economic progress across Chinese provinces, less developed regions often transfer industries to more developed areas, seeking to bolster their own growth trajectories.

Based on the above analysis, we propose the following hypotheses:

H 2a: The total embodied carbon transfer in China’s northern provinces is significantly greater than that in the southern provinces.
H 2b: The carbon embodied by Chinese provinces is mainly transferred to economically developed provinces such as the southern coastal areas.

### 3.3 Decomposition of trade-embodied carbon transfer factors

Existing studies predominantly delve into the determinants of carbon emissions using various research methodologies. For instance, Wang et al. [30] utilized a model capturing the carbon transfers from inter-provincial demands and exports in 2007, 2010, and 2012, incorporating the enhanced log-mean differentiation index decomposition approach. They concluded that the scale effect notably amplifies the net carbon transfers between provinces [30]. Yan et al. [31], integrating both energy and environmental dimensions, constructed a comprehensive "energy-environment-economy" input-output model [31]. Through employing the environmental input-output structural decomposition analysis (EI-SDA) and SPD (structural path decomposition) techniques, they assessed the primary drivers and crucial mitigation pathways for Xinjiang’s energy consumption-induced carbon emissions. Their findings pinpointed the scale effect as both the most pronounced contributor to Xinjiang’s carbon emissions and the most formidable barrier to its reduction. In a separate study, Zhou et al. [2] employed the multi-regional input-output model to evaluate China’s regional economic output from 2002 to 2012. They scrutinized its interregional transfers and pivotal industries and harnessed the structural decomposition analysis method to discern the catalysts behind China’s regional economic activities’ fluctuations. Their results identified the surge in inter-regional trade as the primary engine propelling regional economic cooperation [2]. Meanwhile, Li et al. [32] harnessed both the multi-regional input-output model and the structural decomposition analysis technique to empirically probe into the spatial-temporal evolution and underlying motivators of embodied carbon emissions in Shanxi Province, a key energy hub in China [32]. Our research introduced a nuanced relationship model, aiming to recalibrate the technology and scale of inter-provincial trade, thereby facilitating a more equitable and cost-effective carbon emission reduction.

Based on the above literature analysis, this study put forward the following hypotheses:

H 3: The scale effect has the greatest influence on inter-provincial trade’s embodied carbon transfer.

**Fig 1:**
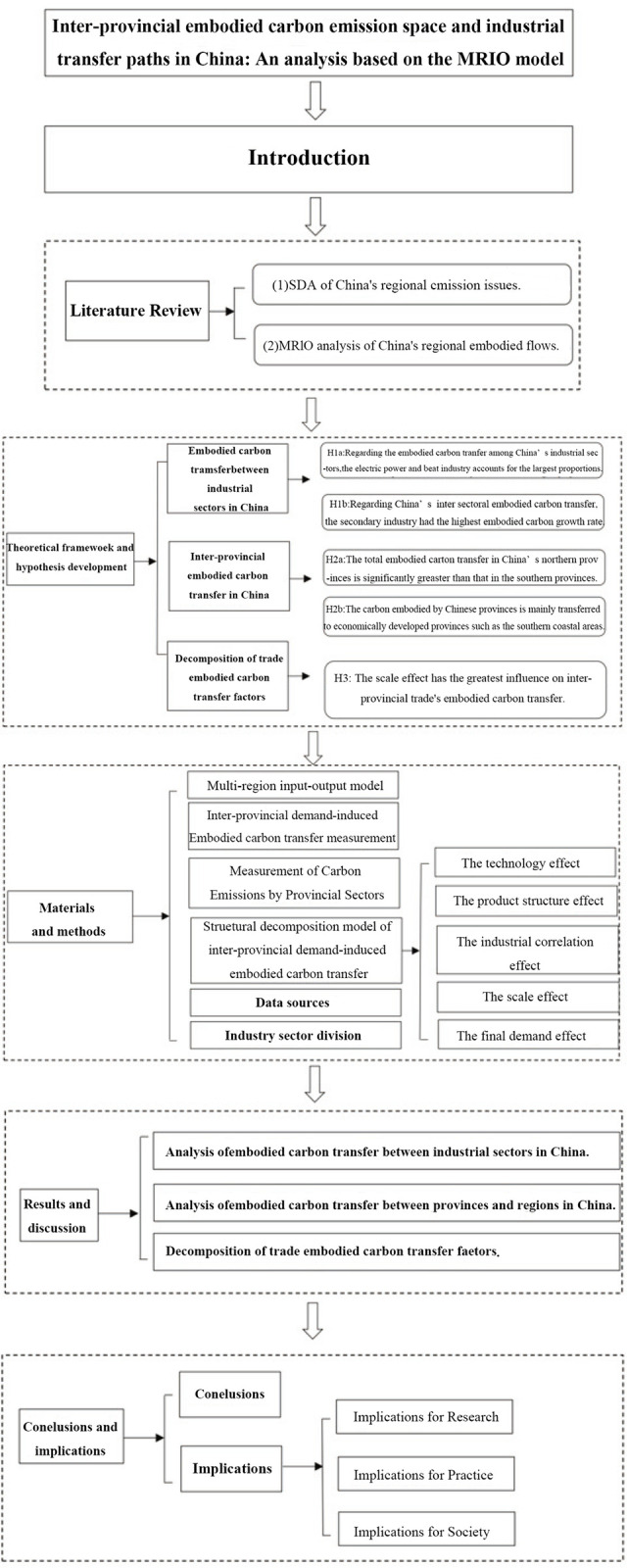
Technical roadmap.

To sum up, this study aims to address three questions: (i) to study the growth patterns spanning industrial sectors and inter-provincial domains as well as the structural shifts in China’s embodied carbon transfers; (ii) to study the trajectories and overarching characteristics of China’s inter-provincial embodied carbon emissions; (iii) to study the determinants of trade’s embodied carbon transfers. As shown below, Fig 1 is the technical roadmap.

## 4 Materials and methods

### 4.1 Multi-region input-output model

The multi-regional input-output model serves as an effective tool for concurrent analysis of carbon transfer and tracing of provincial-level carbon emissions. Initially, the assumptions underlying the multi-regional input-output analysis for China were in alignment with the methodologies presented by Su and Ang [14] and further detailed by Su et al. [33]. Table 1 delineates the structure of the multi-regional input-output table, which has been developed under the non-competitive assumption. It can be seen in the table that the rows and columns of a table represent input and output respectively. In the table, the intermediate transactions and final demands both divide the regions into n sections, from region 1 to region n. And each region could further be divided into section1, section 2…. till section n. So in this table, the compounding data could be divided into $X_{11}^{11}$….to $X_{n}^{m}$. To construct China’s comprehensive multi-regional input-output table, it’s imperative first to procure the input-output tables for each individual province. Subsequently, trade data from these provinces is amalgamated. The matrix for intermediate products is derived from the data concerning inputs from the industrial sectors of each province to their counterparts, inclusive of the originating province. In a similar vein, the matrix for final demand is framed using data on the utilization of the final demand by the industrial sector within every province for every other province, the originating one included. China’s multi-regional input-output, as Table 1, thus, is equipped to provide a nuanced depiction of trade interplays among its diverse provinces.

**Table 1:** Multi-regional input-output table structure imported under the non-competitive assumption.

Input Output			Intermediate							Final							Imports	Total
			Transactions							Demands								Outputs
			region 1	…					region n	region 1	…					region n		
			section	…	section	…	section	…	Section	section	…	section	…	section	…	section		
			1	…	n	…	1	…	N	1	…	n	…	1	…	n		
	region 1	section 1	$X_{11}^{11}$	…	$X_{1n}^{11}$	…	$X_{11}^{1m}$	…	$X_{1n}^{1m}$	$Y_{11}^{11}$	…	$Y_{1n}^{11}$	…	$Y_{11}^{1m}$	…	$Y_{1n}^{1m}$	${EX}_{1}^{1}$	$X_{1}^{1}$
		…	…	…	…	…	…	…	…	…	…	…	…	…	…	…	…	…
		section n	$X_{n1}^{11}$	…	$X_{11}^{1m}$	…	$X_{n1}^{11}$	…	$X_{nn}^{mm}$	$Y_{n1}^{11}$	…	$Y_{11}^{1m}$	…	$Y_{n1}^{11}$	…	$Y_{nn}^{mm}$	${EX}_{n}^{1}$	$X_{n}^{1}$
		…	…	…	…	…	…	…	…	…	…	…	…	…	…	…	…	…
Intermediate		section 1	$X_{11}^{m1}$		$X_{1n}^{m1}$	…	$X_{11}^{mm}$	…	$X_{1m}^{mm}$	$Y_{11}^{m1}$	…	$Y_{11}^{1m}$	…	$Y_{n1}^{mm}$	…	$Y_{1m}^{mm}$	${EX}_{1}^{m}$	$X_{1}^{m}$
Transactions	region n	…	…	…	…	…	…	…	…	…	…	…	…	…	…	…	…	…
		section n	$X_{n1}^{m1}$	…	$X_{mm}^{mm}$	…	$X_{n1}^{mm}$	…	$X_{nn}^{mm}$	$Y_{n1}^{m1}$	…	$Y_{mm}^{mm}$	…	$Y_{n1}^{mm}$	…	$Y_{nn}^{mm}$	${EX}_{n}^{m}$	$X_{n}^{m}$
	import		$I_{1}^{1}$	…	$I_{n}^{1}$	…	$I_{1}^{1}$	…	$I_{n}^{1}$									
	Value Added		…	…	…	…	…	…	…									
	Total Inputs		$X_{1}^{1}$	…	$X_{n}^{1}$	…	$X_{1}^{m}$	…	$X_{n}^{m}$									

In the multi-regional input-output, table structure, formulated under the non-competitive assumption, $X_{ij}^{ab}$ represents the intermediate input from sector *i* at production location *a* to sector *j* at consumption location *b*. $Y_{i}^{ab}$ symbolizes the final product delivered by sector *i* from production region *a* to consumption region *b*, ${EX}_{i}^{a}$ corresponds to the exports of sector *i* from production location *a*, and $X_{i}^{a}$ signifies the total output of sector *i* from production location *a*, where *a* and *b* designate regions, while *i* and *j* denote sectors.

Drawing inspiration from the seminal work of Mose [34], the multi-regional input-output model was leveraged to compute the embodied carbon transfer arising from trade, which can be represented as:

$$X_{i}^{a}=\sum_{j=1}^{n}\sum_{b=1}^{m}X_{ij}^{ab}+\sum_{b=1}^{m}Y_{i}^{ab}+{EX}_{i}^{a}$$ (1)

In the non-competing multi-regional input-output model, the following relationships exist:

$$\left[\begin{array}{c} X_{1}\\ X_{2}\\ \vdots \\ X_{3} \end{array}]=[\begin{array}{ccc} A_{11} & \cdots & A_{1m}\\ \vdots & \ddots & \vdots \\ A_{m1} & \cdots & A_{mm} \end{array}][\begin{array}{c} X_{1}\\ X_{2}\\ \vdots \\ X_{3} \end{array}]+[\begin{array}{c} Y_{11}+Y_{12}+\cdots +Y_{1m}+{EX}_{1}\\ Y_{21}+Y_{22}+\cdots +Y_{2m}+{EX}_{2}\\ \vdots \\ Y_{m1}+Y_{m2}+\cdots +Y_{mm}+{EX}_{m} \end{array}\right]$$ (2)

In Eq (2), X_i_ represents the column vector detailing the total output for each sector within province or city *i*. The matrix A_ij_ illustrates the direct consumption coefficient pertaining to intermediate product inputs transferred from province or city *i* to province or city *j*. Y_ij_ stands for the column vector indicating products from province or city *i* that serve as end-use products for province or city *j*. ${EX}_{i}$ delineates the column vector capturing the exports and imports associated with products from province or city *i*.


$$X_{i}={\left({I-A}_{ii}\right)}^{-1}(\sum_{j=1}^{m}A_{ij}X_{j}+\sum_{j=1}^{m}Y_{ij}+{EX}_{i})\;(j\neq i)$$ (3)

In Eq (3), X_i_ signifies the column vector encapsulating the total output of each sector in province or city *i*. The term ${\left(I-A_{ii}\right)}^{-1}\sum_{j=1}^{m}A_{ij}X_{j}$ (j≠i) quantifies the total output from a specific province or city that’s intended to fulfil the intermediate requirements of other provinces or cities. Similarly, ${\left(I-A_{ii}\right)}^{-1}\sum_{j=1}^{m}Y_{ii}$ (j≠i) measures the complete output of a province or city geared to address the final product demands of other provinces or cities. The component ${\left(I-A_{ii}\right)}^{-1}\sum_{j=1}^{m}Y_{ii}$ represents the total output of a province or city aimed at meeting its own final product requirements. Lastly, (I−A_ii_)^−1^EX_i_ characterizes the total output from the province or city designated to accommodate export necessities.

### 4.2 Inter-provincial demand-induced Embodied carbon transfer measurement

In the realm of multi-regional embodiment analysis, two predominant methods are employed: MRIO (Multi-Regional Input-Output) and EEBT (Emissions Embodied in Bilateral Trade). The Emissions Embodied in Bilateral Trade approach recalibrates "production-oriented" emissions by considering the emissions embodied in bilateral trade, which in turn provides a perspective on "consumption-oriented" emissions. A notable distinction of the Emissions Embodied in Bilateral Trade method is its lack of differentiation between emissions from imported goods used for intermediate versus final consumption. Instead, it directly attributes these emissions to the importing country. While the Emissions Embodied in Bilateral Trade approach offers transparency, making it particularly advantageous for analysing national trade and climate policies, the multi-regional input-output method brings an added dimension. It accommodates the allocation of emissions present in intermediate consumption to the nations that consume them. Further, multi-regional input-output encapsulates information about the economic structure and inter-regional relationships, providing a holistic view. Drawing from Su and Ang’s work [33], we can define $\hat{Q_{i}}$ as the diagonal matrix representing carbon emission intensity factors per unit of total output for industry i, whether in a province or a city. Following the framework put forth by Xia et al. [35], the column vector detailing carbon emissions for each industrial sector within a region can be described as follows:

$$\overset{⏜}{Q_{l}}X_{i}=\overset{⏜}{Q_{l}}{\left({I-A}_{ii}\right)}^{-1}(\sum_{j=1}^{m}A_{ij}X_{j}+\sum_{j=1}^{m}Y_{ij}+\overset{⏜}{Q_{l}}X_{i})\;(j\neq i).$$ (4)

In Eq (4), ${\hat{Q_{i}}X}_{i}$ symbolizes the column vector detailing carbon emissions for each industrial sector within a specific region. X_i_ represents the column vector indicating the complete output of each sector within province or city i. The term ${\left(I-A_{ii}\right)}^{-1}\sum_{j=1}^{m}A_{ij}X_{j}$ (j≠i) quantifies the aggregate output from a specific province or city that’s intended to cater to the intermediate demands of other provinces or cities. Similarly, ${\left(I-A_{ii}\right)}^{-1}\sum_{j=1}^{m}Y_{ij}$ (j≠i) illustrates the comprehensive output of a province or city devised to meet the end-product requirements of other provinces or cities. The component ${\left(I-A_{ii}\right)}^{-1}\sum_{j=1}^{m}Y_{ii}$ portrays the total output of a province or city earmarked to serve its own end-product necessities. Lastly, ${\left(I-A_{ii}\right)}^{-1}{EX}_{i}$ captures the complete output from the province or city designated for export needs.

When considering only domestic trade, the model for embodied carbon transfer between production location *a* and consumption location *b* is articulated as:

$${EMC}_{ab}=\hat{Q_{a}}{\left(I-A\right)}^{-1}(A_{ab}X_{b}+Y_{b})$$ (5)

In Eq (5), $\hat{Q_{a}}$ signifies the diagonalized carbon intensity factor matrix pertaining to province or city a. *EMC*_*ab*_ denotes the embodied carbon emissions resulting from the consumption of end products of province or city *b*, which is channelled through the industrial supply chain to province or city *a*. This is effectively illustrative of the trade-embodied transfers of carbon emissions (TCEs) from province or city *a* to province or city *b*.

### 4.3 Measurement of carbon emissions by provincial sectors

The formula for calculating carbon emissions of provincial departments, based on the estimation method of the Intergovernmental Panel on Climate Change (IPCC), is as follows:

$${TCE}_{i}=\sum_{k}{TCE}_{ik}=\sum_{k}{FF}_{ik}*{COR}_{ik}*{LCV}_{ik}*{LCC}_{ik}*44/12$$ (6)

In the above equation, *TCE*_*i*_ denotes the total emissions from energy consumption of department *i*, which is the sum of the carbon dioxide emissions from *k* types of fossil fuels, represented as *TCE*_*ik*_, *FF*_*ik*_ and *LCC*_*ik*_ refer to the consumption and carbon oxidation rate of fossil fuel *k* for department *i*, respectively. *COR*_*ik*_ and *LCV*_*ik*_ respectively represent the average calorific value and carbon content of fossil fuel *k*, The factor 44/12 indicates the molar ratio of carbon dioxide to carbon.

### 4.4 Structural decomposition model of inter-provincial demand-induced embodied carbon transfer

The Structural Decomposition Analysis (SDA) method is deeply rooted in the input-output model, a tool frequently employed to investigate the determinants influencing the temporal growth of embodied carbon emissions in trade. In this context, the cumulative embodied carbon emissions from trade can be conceptualized as a multiplicative outcome of several contributing factors. Specifically, the growth trajectory of trade-embodied carbon emissions can be dissected into the sum of distinct independent variables. The impact of each variable on the growth of these emissions is gauged by observing variations in these independent components. To enhance the precision of model measurements and in alignment with the methodology of Su and Ang [14] this research leans on the widely-accepted two-stage approach for decomposing the evolution of trade-embodied carbon emissions via structural decomposition analysis.

Tracing its origin to the pioneering work of Leontief and colleagues, the structural decomposition analysis methodology aims to quantify the contribution of each independent variable to shifts in a dependent variable. This is accomplished by parsing the change in the dependent variable within an economic framework into an aggregation of alterations in the pertinent independent variables. Within this context, the embodied carbon values for the reference and base periods are delineated as *EMC*(*t*) and *EMC*(0) respectively. The differential, ΔEMC=EMC(t)−EMC(0), encapsulates the shift in embodied carbon from the baseline to the reference timeframe.

To circumvent issues of non-uniqueness inherent to structural decomposition analysis application, our analysis adopts a bi-level decomposition, comparing reference and baseline periods. By averaging these, we derive the subsequent equations:

ΔEMC=f(ΔE)+f(ΔL)+f(ΔA)+f(ΔX)+f(ΔY) (7)

$$\Delta EMC=\frac{1}{2}[\Delta EL(0)A(0)X(0)Y(0)+\Delta EL(t)A(t)X(t)Y(t)]+\frac{1}{2}[E(t)\Delta LA(0)X(0)Y(0)+E(0)\Delta LA(t)X(t)Y(t)]+\frac{1}{2}[E(t)L(t)\Delta AX(0)Y(0)+E(0)L(0)\Delta AX(t)Y(t)]+\frac{1}{2}[E(t)L(t)A(t)\Delta XY(0)+E(0)L(0)A(0)\Delta XY(t)]+\frac{1}{2}[E(t)L(t)A(t)X(t)\Delta Y+E(0)L(0)A(0)X(0)\Delta Y].$$ (8)

$f(\Delta E)=\frac{1}{2}[\Delta EL(0)A(0)X(0)Y(0)+\Delta EL(t)A(t)X(t)Y(t)]$ delineates the effect of changes in carbon emission intensity on embodied carbon transfer, commonly referred to as the technology effect.

$f(\Delta L)=\frac{1}{2}[E(t)\Delta LA(0)X(0)Y(0)+E(0)\Delta LA(t)X(t)Y(t)]$ conveys the influence that product structure has on embodied carbon transfer, termed the product structure effect.

$f(\Delta A)=\frac{1}{2}[E(t)L(t)\Delta AX(0)Y(0)+E(0)L(0)\Delta AX(t)Y(t)]$ captures the repercussions of direct consumption coefficients on the interrelationships both preceding and succeeding industries, denominated as the industry linkage effect. Expanding on the industry linkage effect, the notion of industrial correlation suggests that an industry’s economic activities—encompassing production, technology, and more—can shape the economic undertakings of its adjacent sectors. Based on this conceptualization, the industry correlation effect bifurcates into forward and backward correlation impacts.

$f(\Delta X)=\frac{1}{2}[E(t)L(t)A(t)\Delta XY(0)+E(0)L(0)A(0)\Delta XY(t)]$ embodies the influence of the aggregate inter-provincial trade output size on embodied carbon transfer, otherwise described as the scale effect.

$f(\Delta Y)=\frac{1}{2}[E(t)L(t)A(t)X(t)\Delta Y+E(0)L(0)A(0)X(0)\Delta Y]$ characterizes the ramifications of final demand on the embodied carbon transfer across provinces and cities, aptly termed the final demand effect.

### 4.5 Data sources

The primary objective of this study was to quantify the transfer of embodied carbon emission flows among provinces. Our main data sources encompassed national value-based multi-regional input-output table data and provincial carbon emission inventories. Due to data constraints related to Hong Kong and Macao, the scope of this investigation was limited to the 31 mainland provinces of China. The primary sources for the measurement data of carbon emissions for provincial departments are the "General Rules for Comprehensive Energy Consumption Calculation" (GB/T2589-2008) proposed by the National Development and Reform Commission (NDRC) and the National Standards Committee, the "2006 Intergovernmental Panel on Climate Change Guidelines for National Greenhouse Gas Inventories", and the "Guidelines for Compiling Provincial Greenhouse Gas Inventories.

### 4.6 Industry sector division

In the national multi-regional input-output tables for the years 2012, 2015, and 2017, the division of industrial sectors did not align with the classification present in the local carbon emission inventory. To ensure consistency, we consolidated the 42 industrial sectors from the provinces into 28 sectors. This reclassification facilitates a unified measurement across industries. It’s important to note that due to statistical limitations, our calculations also incorporate CO2 emissions from both Hong Kong and Macao.

## 5 Results and discussion

China’s inter-provincial trade has displayed a growing trend in terms of embodied carbon transfer. Specifically, the total embodied carbon transfers were recorded at 4697.65 million tons in 2012, advancing to 5078.66 million tons in 2015, and reaching 5256.86 million tons in 2017. Over a span of five years, this represented an increase of 11.90%, equivalent to an augmentation of 559.21 million tons. However, when viewed longitudinally, certain provinces or industries within China exhibited a declining trend in their embodied carbon transfers.

### 5.1 Analysis of embodied carbon transfer between industrial sectors in China

An in-depth examination of the embodied carbon transfer characteristics across 2012, 2015, and 2017, categorized by industrial sectors, elucidated that the secondary and tertiary industries dwarfed the primary industry in terms of embodied carbon transfers. Specifically, sectors such as electricity and heat generation and supply; metal smelting and calendaring products; non-metallic mineral products; transportation, storage, and postal services; coal mining products; petroleum, coking, and nuclear fuel processing products; and chemical products emerged as pivotal drivers of economic shifts, thereby validating Hypothesis H1a. These observations align with the findings of Li [32], who identified industries with pronounced net embodied carbon outflows, prominently featuring sectors like electricity and heat production and supply, metal smelting and rolling processing, and petroleum processing, coking, and nuclear fuel processing.

This prominence can be attributed to two primary factors. Firstly, embodied carbon emissions encapsulate both direct and indirect measurement dimensions. The intricate supply chain of heavy industries, replete with myriad intermediate production stages, leads to escalating embodied carbon emissions. Secondly, the significant fossil energy consumption of heavy industries inherently results in elevated embodied carbon emissions. Given that heavy industry stands as the primary energy consumer across Chinese regions, it inherently becomes the central source of embodied carbon emissions. Hence, amplifying its energy efficiency and moderating its emission intensity is paramount.

**Fig 2:**
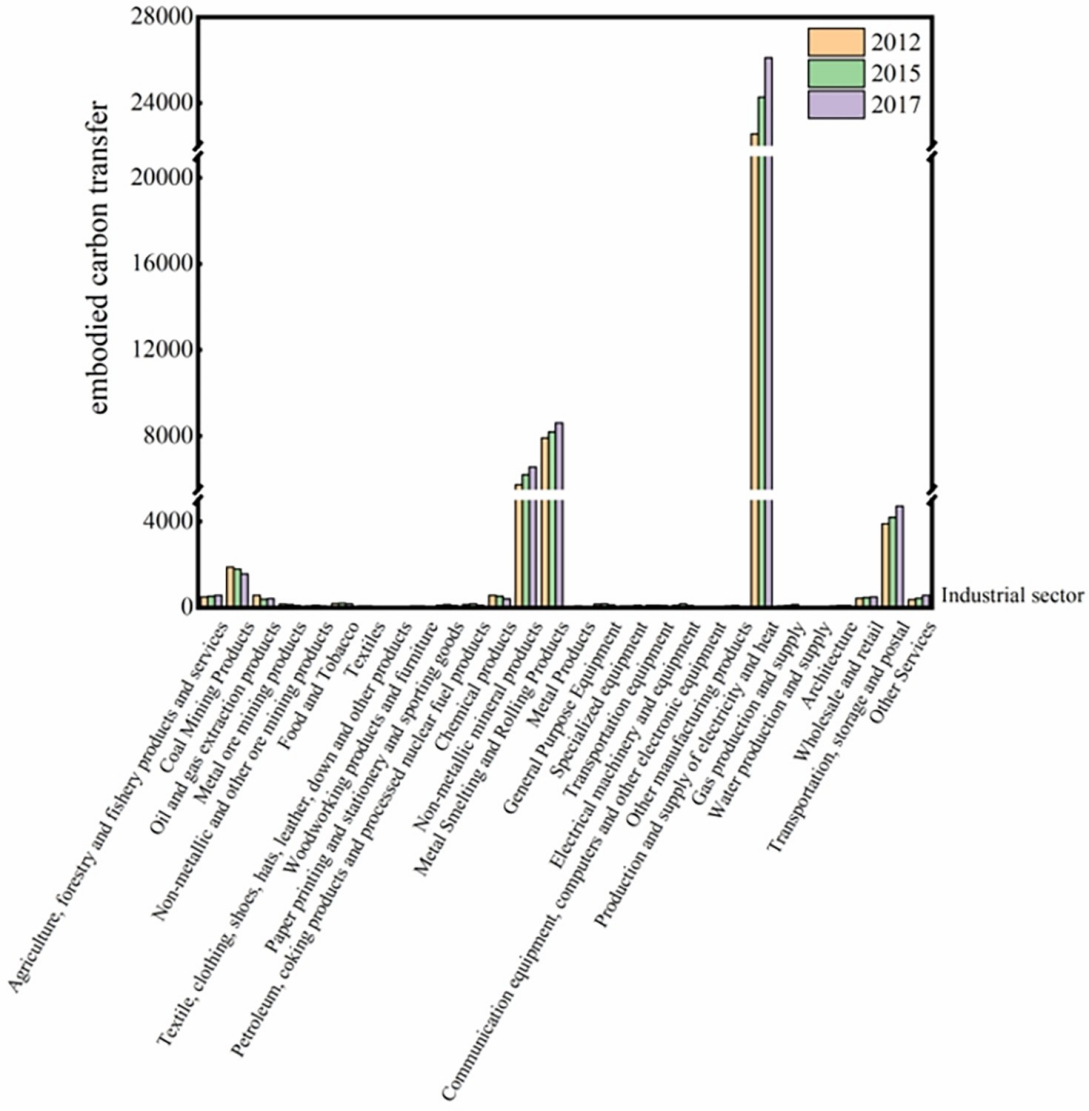
Trend of embodied carbon transfers across 28 Industries for the years 2012, 2015, and 2017(million tons). Note: Derived from the data calculation. Refer to section 4.3 for the calculation method.

Such costs are intrinsically tied to carbon emissions stemming directly from the industrial sector and auxiliary production and processing activities. For instance, the generation and supply of electricity and heat involve significant coal combustion and other energy sources, subsequently leading to heightened carbon emissions. Within the industrial realm, the secondary industry reigns supreme as the principal carbon transfer emitter, whereas the primary and tertiary sectors exhibit subdued dominant carbon transfers. Concurrently, the chemical segment of the secondary industry also portrayed elevated carbon transfers, with the carbon transfer ratios of coal extraction products, chemicals, petroleum, coking, and nuclear fuel processing product industries being notably substantial, as illustrated in Fig 2. Grounded in the empirical evidence presented above, Hypothesis H2b was examined. In the pursuit of sustained economic growth, China’s trajectory of accelerating industrialization is inevitable. Historically, industries are notorious for their significant energy consumption and resultant emissions. Furthermore, as urbanization levels continue to soar, the ensuing demand for infrastructure and transportation is poised to burgeon, thereby propelling industrial production and, by extension, exacerbating carbon emissions from the secondary industry, rendering them significantly higher than those from the primary and tertiary sectors.

Given the pronounced disparities in embodied carbon emissions between the electricity and heat production sectors and other industries, this study adjusts the vertical axis for embodied carbon emissions. This modification enables a clearer and more direct representation of temporal variations in embodied carbon emissions across different industrial sectors.

In 2012, the electricity and heat production and supply sector dominated embodied carbon transfers, accounting for 50.15% of the total, which equated to a substantial 2355.86 million tons. Metallurgical smelting and rolling processes contributed 16.84% with a volume of 791.13 million tons, while non-metallic mineral products constituted 12.19%, totalling 572.95 million tons. Fast forward to 2015, the electricity and heat sector’s share slightly increased to 51.73% (2627.32 million tons), and by 2017, it further edged up to 51.84%, corresponding to 2725.18 million tons. Evaluating the embodied carbon transfer volumes across 2012, 2015, and 2017, it becomes apparent that electricity and heat generation and supply emerge as the predominant sector.

Such predominance stems from the intertwining dynamics of industrial production and overarching national policies, notably China’s West-to-East Power Transmission initiative which facilitates inter-provincial electricity transmission. While China’s direct carbon emissions have witnessed a downtrend, the embodied carbon transfers within industry chains remain staggering. This underscores the imperative for China to not only curtail direct-source carbon emissions, like those from factories, but to also channel investments into technological enhancements in industrial fabrication and processing. Aiming to reduce embodied carbon emissions at the granular, technical level, strategies like Carbon Capture, Utilization, and Storage (CCUS) technology become essential. Consequently, a shift towards cleaner energy substrates is vital. Although electricity is heralded as a clean energy conduit, the prevailing power blend remains heavily skewed towards thermal power generation, which relies extensively on fossil-based resources, predominantly coal, during production.

**Fig 3:**
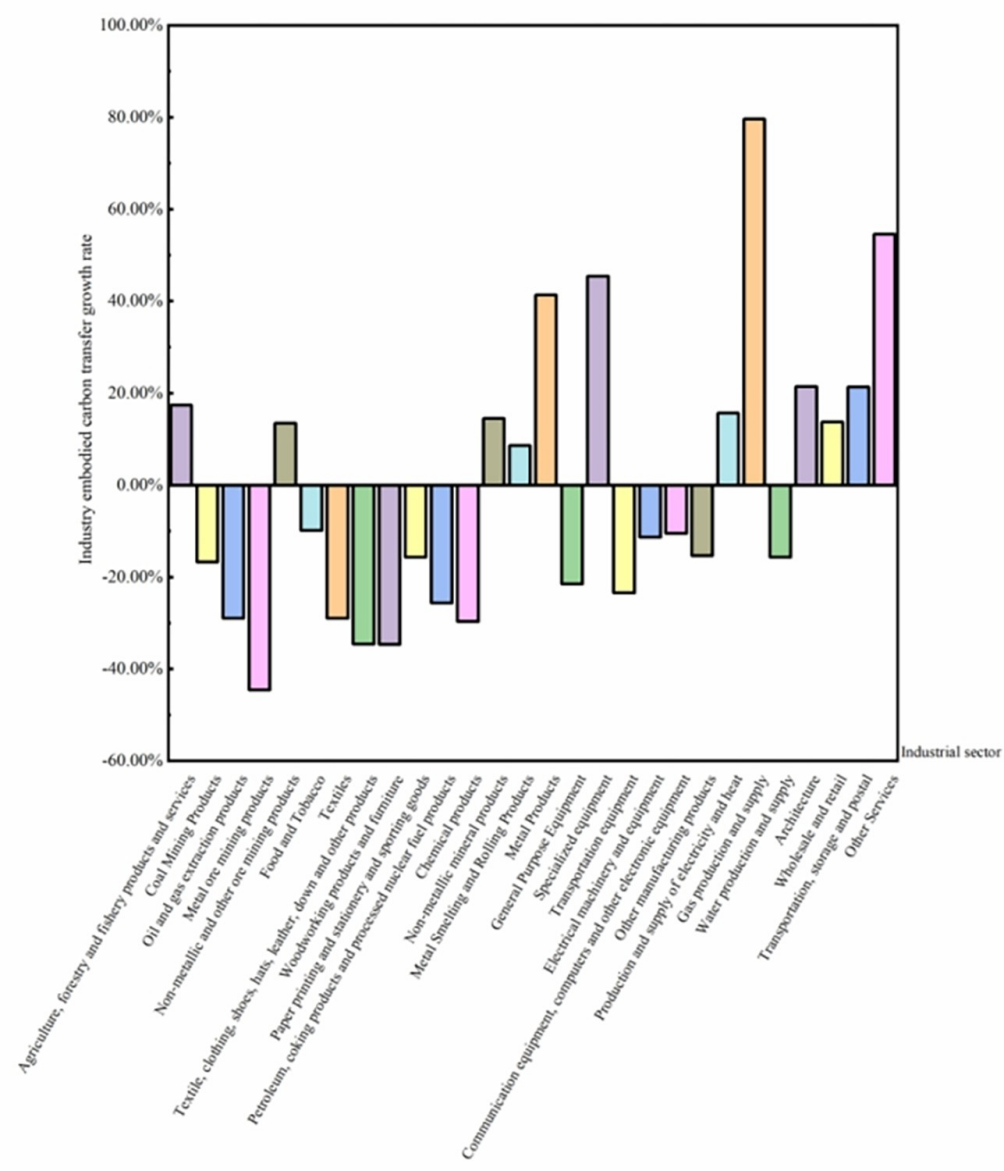
Growth rate of embodied carbon transfers across 28 industries from 2012 to 2017. Note: Data calculated based on Fig 2.

When amalgamating the embodied carbon transfer share of the industry across the years 2012, 2015, and 2017, the production and supply of electricity and heat observed a discernible augmentation with a growth rate of 15.67%. In contrast, the cumulative growth rate for trade-embodied carbon transfer stood at 11.90%. A sector-wise analysis, as depicted in Fig 3, highlighted that the gas production and supply sector experienced a surge at 79.65%, even though its overall contribution to embodied carbon transfer was modest. Following closely, other services, specialized equipment, and metal products sectors registered substantial growth rates of 54.57%, 45.46%, and 41.34%, respectively.

Conversely, the chemical industry’s growth outpaced that of coal mining products, and oil and gas extraction products, which witnessed negative growth rates of -16.73%, -28.96%, and -29.65% respectively. A noteworthy observation is that some industrial sectors with substantial transfer flows experienced negative growth, while industries with more moderate transfer magnitudes recorded considerably higher growth rates.

Further, the tertiary sector registered an elevated embodied carbon growth rate, likely indicative of shifts in China’s consumption patterns. One plausible reason could be the substantial carbon footprints from the tourism sector, primarily stemming from tourist transportation. Given the considerable distances tourists traverse from their origin to destination, substantial fossil fuel consumption ensues. Within the tertiary realm, the service industry, characterized by a dense population, implies that population metrics play a pivotal role in influencing the sector’s carbon emissions. Concurrently, the petrochemical sector manifested significant emission curtailments, resonating with China’s policy-driven ambitions of attaining carbon peak and neutrality.

**Fig 4:**
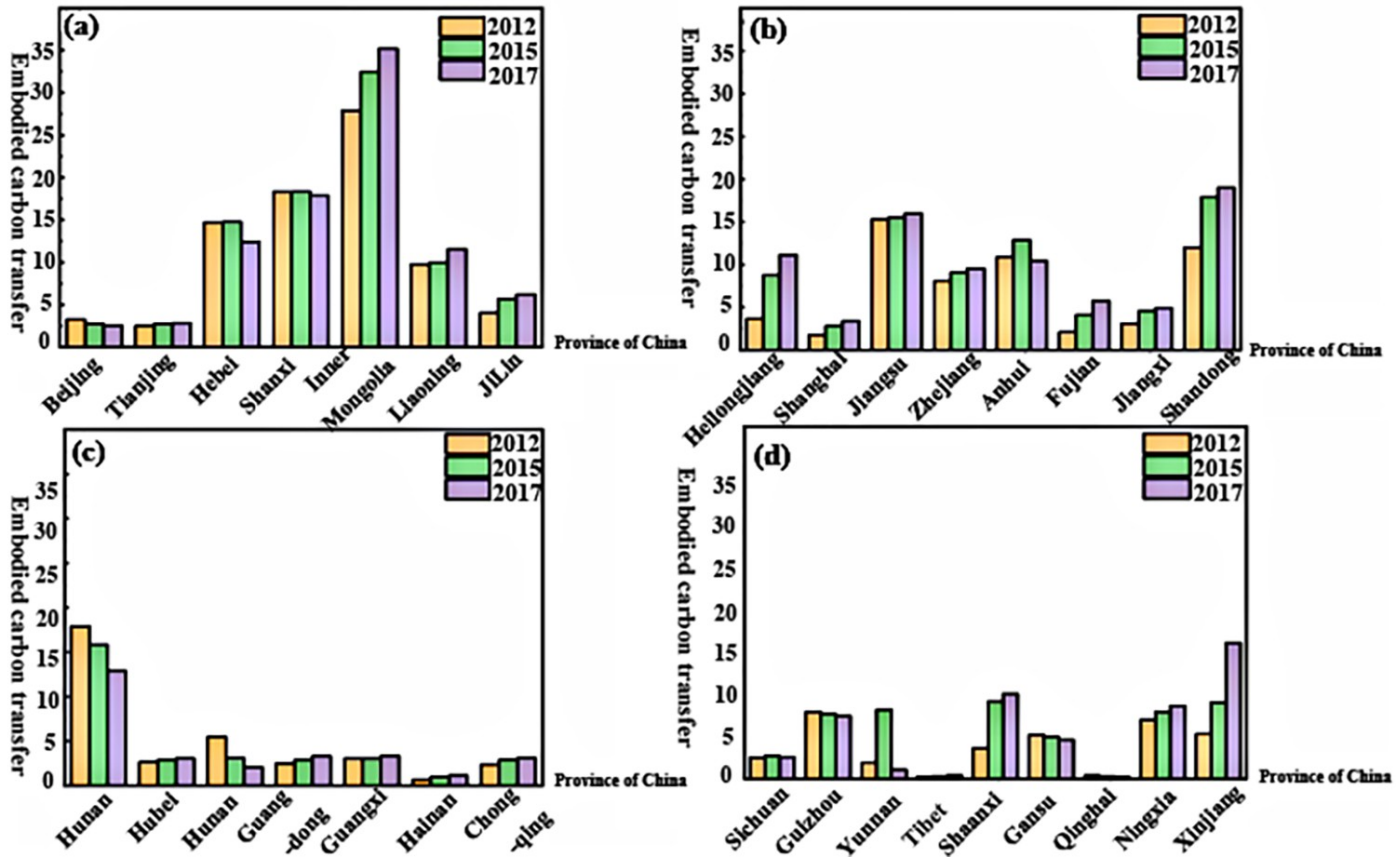
(a-d) Trends in Inter-provincial Embodied Carbon Transfer for the Production and Supply of Electricity and Heat across China’s 31 Provinces for 2012, 2015, and 2017(10 million tons). Note: Data derived from calculations. See method in section 4.3.

In our analysis, we gave prominence to the electricity and heat industry’s generation and supply sectors, as they accounted for over 50% of the embodied carbon transfer. Fig 4 delineates that larger transfers are evident in energy-centric provinces such as Inner Mongolia, Shanxi, and Hebei. Intriguingly, economically affluent coastal and central provinces also exhibited significant inter-provincial embodied carbon transfers, mirroring the patterns found elsewhere. The prevalent energy structure in these regions heavily leans towards coal. Consequently, their energy consumption and carbon emissions bear a striking resemblance, emanating from the same foundational sources, processes, and origins, further underscoring the intricacies of regional carbon exchanges.

### 5.2 Analysis of embodied carbon transfer between provinces and regions in China

**Fig 5:**
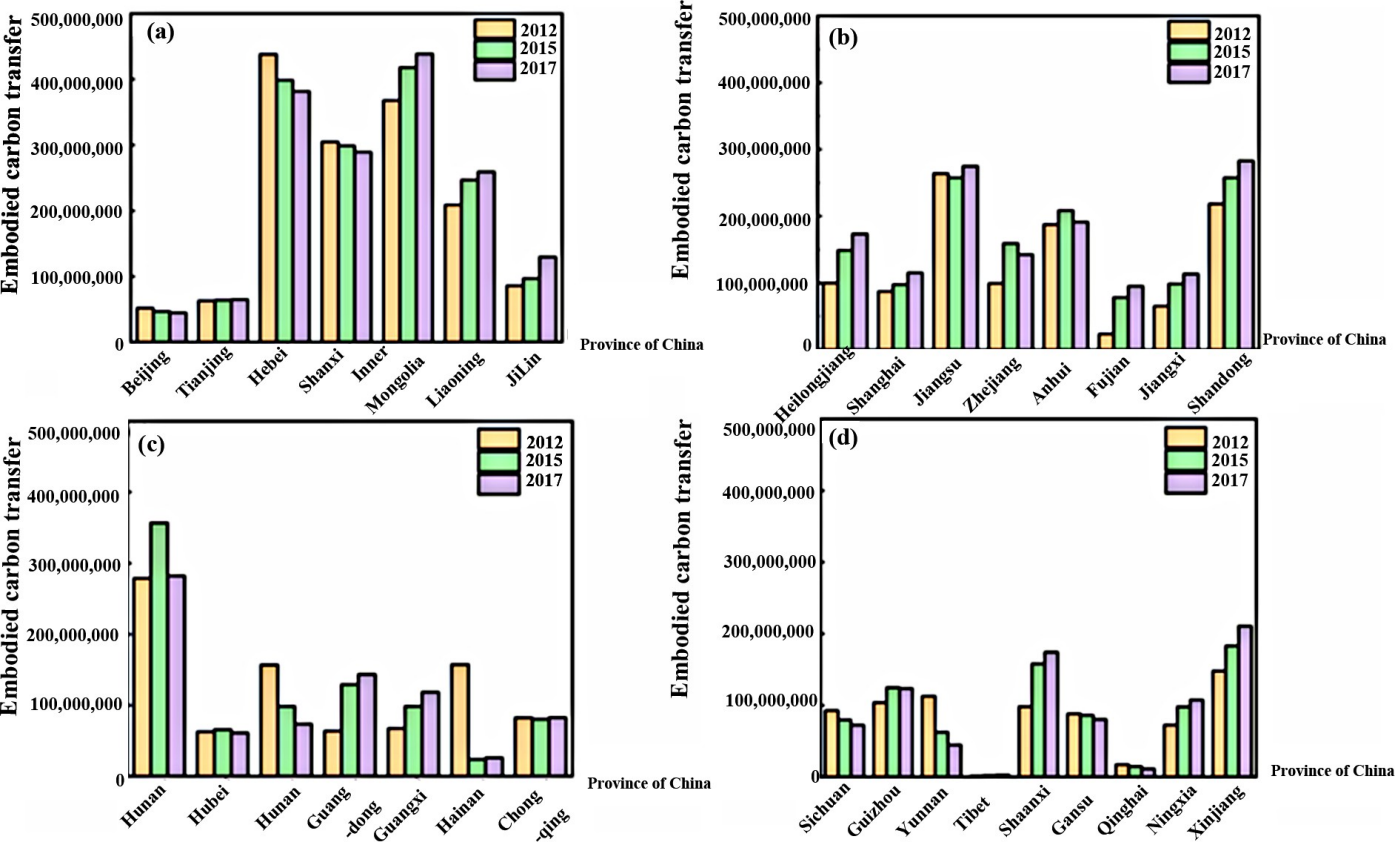
(a-d) Trends of Embodied Carbon Transfer across China’s 31 Provinces in 2012, 2015, and 2017(10 tons). Note: Data derived from calculations. See method in section 4.2.

As elucidated in Fig 5, at the inter-provincial level, trade’s embodied carbon transfer for the years 2012, 2015, and 2017 was predominantly elevated in energy-centric provinces like Hebei, Inner Mongolia, and Shanxi. Their respective totals were 437.8, 398.7, and 381.7 million tons; 367.8, 417.9, and 438.4 million tons; and 304.8, 298.7, and 289.2 million tons. The distinctive resource endowments of these energy-focused provinces have inherently amplified their embodied carbon transfers in trade. This observation aligns with the findings of Wu et al. [36] and Wang et al. [30], who asserted that coastal and headquarters provinces witness heightened embodied carbon transfers.

Furthermore, the embodied carbon transfers were particularly pronounced in several coastal and central regions, including Jiangsu, Zhejiang, Shandong, and Henan. These provinces, having undergone accelerated economic growth, manifest demand-driven embodied carbon emissions. Their strategic positioning as transportation hubs facilitates expansive trade flows, which in turn, escalates embodied carbon transfers. On the other hand, regions like Xinjiang, Qinghai, Yunnan, and Hainan registered relatively modest embodied carbon transfers, attributable to variances in geographical placement, economic development trajectories, and resource availabilities.

Considering the overarching trends, northern provinces of China experienced a more significant embodied carbon transfer compared to their southern counterparts. The insights derived from these results provide a basis to evaluate Hypotheses H2a and H2b.

**Fig 6:**
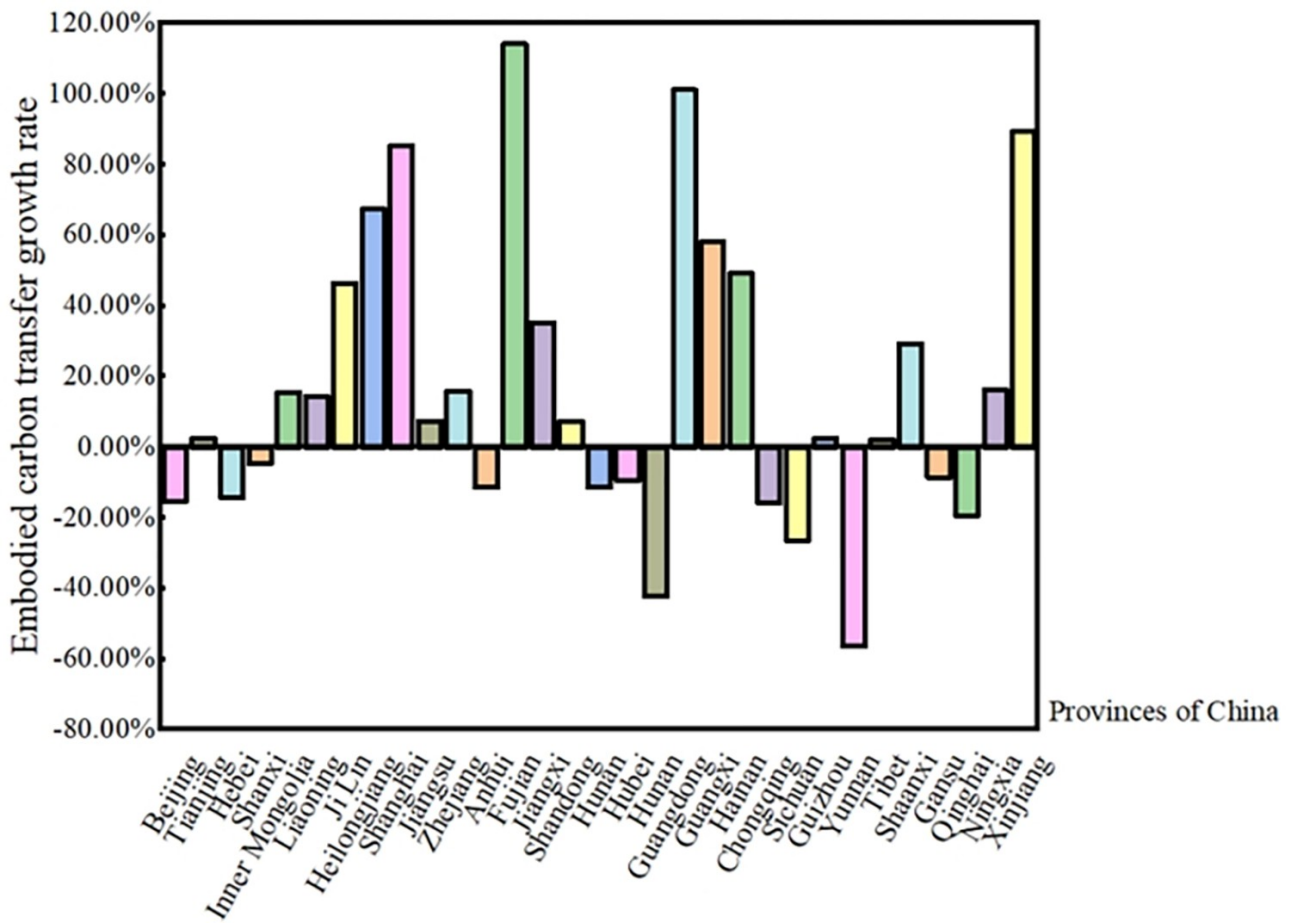
Growth rate of embodied carbon transfer across China’s 31 provinces from 2012 to 2017. Note: Data calculated based on Fig 5.

As depicted in Fig 6, the surge in embodied carbon transfer from 2012 to 2017 was notably pronounced in certain provinces, including Fujian, Guangdong, Shanghai, and Xinjiang. These provinces recorded growth rates of 114.21%, 101.24%, 85.43%, and 89.32% respectively. Intriguingly, most provinces that exhibited significant growth rates initially had relatively modest totals of embodied carbon transfers. Yet, accelerated economic development, advancements in technology, or strategic policy interventions spurred a rapid surge in trade-related embodied carbon transfers. A case in point is Xinjiang, where wind power production witnessed a meteoric rise within a mere five-year span. Likewise, the economies of both Guangdong and Shanghai saw substantial growth.

Contrastingly, several provinces, despite having considerable embodied carbon transfers, displayed negative growth rates. This can be attributed to a series of factors. For instance, factories with high carbon footprints might have relocated outside the province. Policy initiatives, in alignment with China’s commitment to green development, mandated certain provinces like Hebei and Shanxi to curtail their emissions. It’s evident that economically affluent provinces reap the benefits of low-carbon gains from interregional trade, influencing the carbon emission dynamics of the region. Concurrently, less economically advanced regions bear the onus of counterbalancing carbon emissions from their wealthier counterparts.

**Fig 7:**
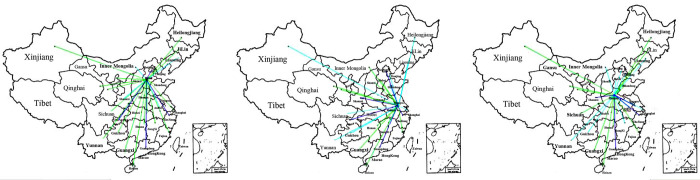
Flow diagram of embodied carbon transfer for China’s 30 provinces and cities from Henan, Jiangsu and Hebei provinces. Note: Data derived from calculations. Refer to section 4.2 for the calculation method. The figure was created by the author, with copyright information available at: http://bzdt.ch.mnr.gov.cn/.

The intricacies of embodied carbon transfer networks between provinces present a considerable challenge when attempting to delineate their transfer characteristics on a singular map. Consequently, representing every province could be somewhat redundant. To streamline the analysis, this study opted to focus on key representative provinces, such as Hebei, a resource-driven province. We examined Hebei’s embodied carbon transfer patterns, as depicted in Fig 7. In this figure, the varying shades and line thicknesses symbolize the spectrum of embodied carbon from high to low: the embodied carbon transfer between Hebei and Beijing, Chongqing, Guangdong and Zhejiang Province were high. By contrast, the embodied carbon transfer between Hebei and Inner Mongolia, Shaanxi and Anhui were low. Other provinces such as Qinghai, Guizhou and Shandong witness a medium carbon transfer with Hebei Province. In terms of Jiangsu, we can find that the embodied carbon transfer between it and Beijing, Chongqing, Zhejiang, Shaanxi and Guangdong is high while the relationship between it and Heilongjiang, Xinjiang, Tianjin, Guizhou, Yunnan, Shanghai and Sichuan is relatively low. As is shown in the third figure in Fig 7, for another province Henan, a high carbon transfer between it and Shaanxi, Zhejiang, Guangdong and Jiangsu is high while its correlation between Inner Mongolia, Guizhou and Yunnan is low.

Fig 7 reveals that at the provincial level, embodied carbon predominantly gravitated towards economically developed provinces, notably those along the southern coast. Additionally, consistent with spatial distribution traits, provinces exhibited a stronger propensity to transfer embodied carbon to their immediate neighbours. The southern coastal region, with its advanced economy, attracts residents with elevated living standards and heightened environmental consciousness. This dynamic amplifies the efficiency of embodied carbon transfer and optimizes energy utilization. The economic disparities among Chinese provinces have birthed distinct regional economic and ecological ambitions, granting businesses opportunities to seek regions with laxer environmental constraints, or "pollution paradises."

In the realm of regional trade, geographical proximity and streamlined transportation infrastructure offer neighbouring provinces a spatial edge in trade fluxes. From a policy-driven standpoint, certain provinces are funnelled into trade dependencies with specific energy-rich provinces; for instance, the relocation of many Beijing-based factories to Hebei, escalating the embodied carbon transfer between the two provinces. Such transfers exhibit spatial correlations, reflecting both geographical immediacy and complementarity of industrial structures. Examining it from a final demand perspective, provinces with expansive consumption profiles tend to break geographical barriers in pursuit of rapid economic expansion. As the added value from intermediate and final products transitions, a more substantial chunk of trade-embodied carbon infiltrates other regions, which consequently shoulder heightened embodied carbon emissions. It’s particularly noteworthy that the carbon transfer to Guangdong from the three provinces was exceptionally robust, highlighting Guangdong’s expansive trade and consumption patterns which surpass regional confines. Factors such as population migration from Henan to Guangdong and the influx into the tertiary sector significantly amplified the embodied carbon transfer dynamics. Consistent with Wu [36], the study observed a trend wherein embodied carbon emissions drift from economically buoyant provinces towards resource-abundant, heavy-industry-focused areas, navigating from the southern to northern regions and from the coast to the inland.

### 5.3 Decomposition of trade embodied carbon transfer factors

**Fig 8:**
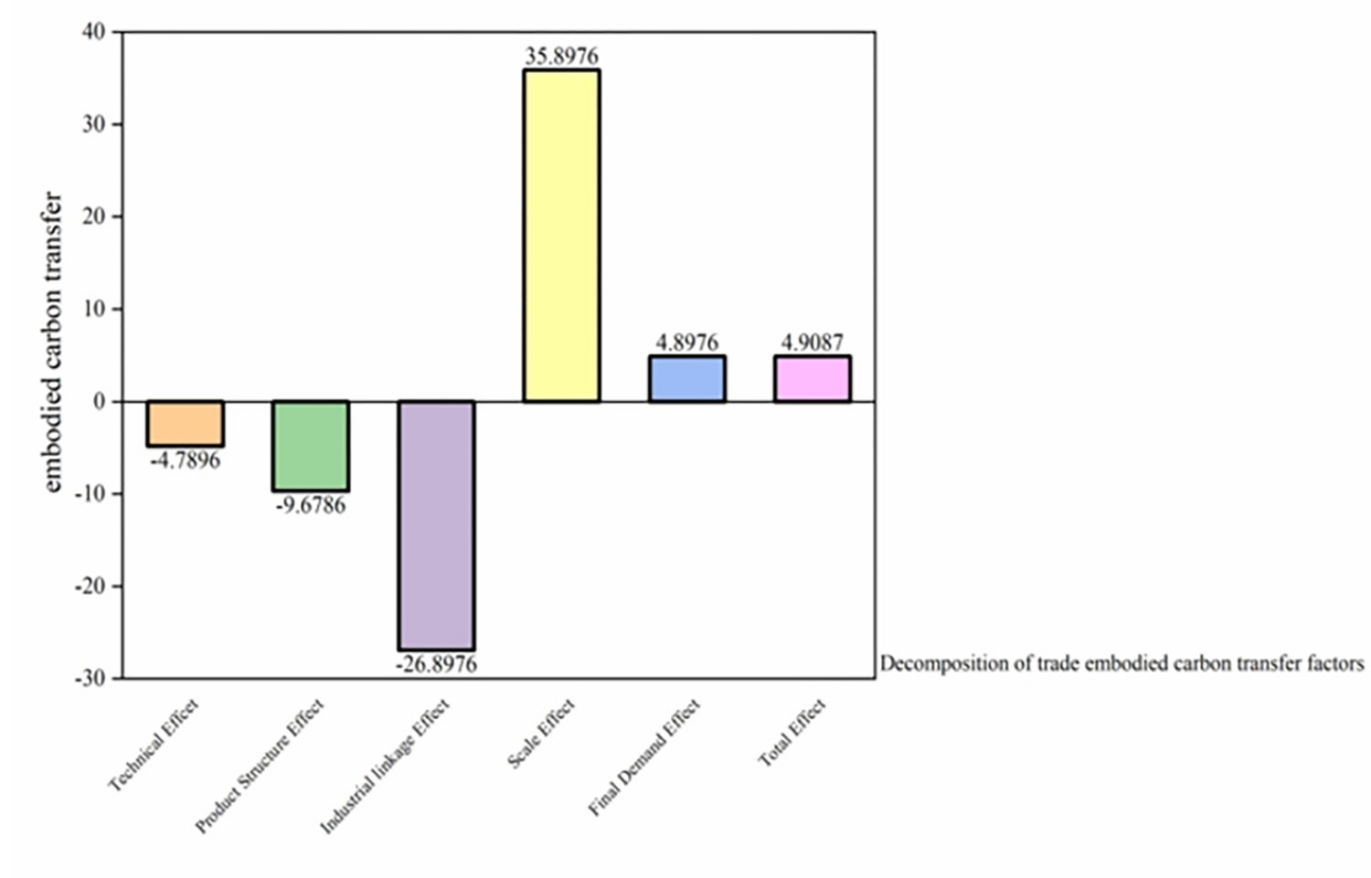
Embodied carbon emissions from the technology effect, the product structure effect, the scale effect, the industrial linkage effect, and the final demand effect (100 million tons). Note: Data derived from calculations. Refer to section 4.4 for the calculation method.

As depicted in Fig 8, of the five influential factors considered, the scale effect was the most pronounced. It resulted in an increase in embodied carbon transfer from interprovincial trade by 3589.76 million tons, thus corroborating H3. Conversely, the industry linkage effect diminished the embodied carbon transfer from inter-provincial trade by 2689.76 million tons. The technological and product structure influences further reduced the inter-provincial embodied carbon transfer by 478.96 and 967.86 million tons, respectively.

Such outcomes underscore that the sheer magnitude of China’s interprovincial commerce, paired with its brisk economic ascent, has amplified the trade scale, subsequently elevating carbon emissions. Besides refining China’s provincial industrial configuration, enhancements in inter-industry production and processing chains have considerably mitigated carbon emissions. As alluded to earlier, the study delved into the mechanisms via which each factor impacts carbon emissions, both at the inter-provincial and industrial sectoral levels. The aim was to discern the primary catalysts for carbon emissions. Broadly, technological advancements across provinces and cities were predominantly evident at the inter-provincial dimension, curtailing embodied carbon transfer during the production phase.

**Fig 9:**
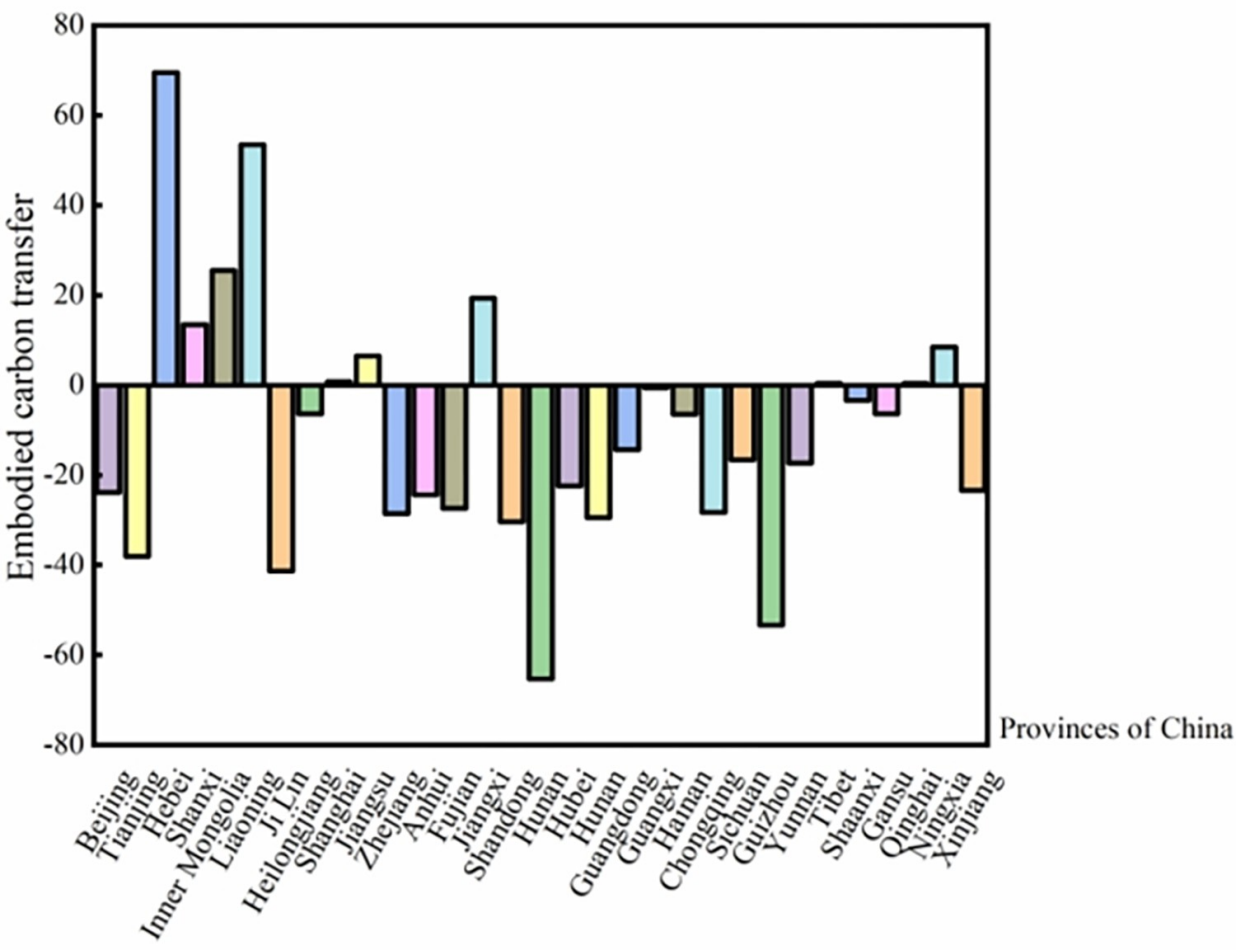
Inter-provincial embodied carbon transfer technology effect across China’s 31 provinces (million tons). Note: Data derived from calculations. See method in section 4.4.

As depicted in Fig 9, technological advancements lead to a decline in carbon emission intensity, positioning the technology effect in the negative domain and signifying efficient energy utilization by the province or city in question. The daily adoption of cleaner production techniques is on the rise, with the carbon-reducing impact of technological advancements surpassing that of shifts in industrial structures. Furthermore, these technological strides not only diminish a region’s carbon emissions but also have a spillover effect, curtailing carbon emissions in neighbouring areas. Thus, technological advancement stands as a pivotal determinant in industrial pollution abatement.

However, provinces like Hebei, Shanxi, and Inner Mongolia displayed a positive technical effect. Such energy-centric provinces, endowed with distinct resources, face challenges in reducing carbon emissions during resource extraction and utilization, unlike other economically-focused provinces and cities. This challenge correlates with the stagnation in advancing resource extraction processes; for instance, coal mining in Shanxi hasn’t embraced significant technological innovation—a widespread issue internationally. The scope of technological innovation in these regions lags in aspects like efficient coal use, energy conservation, environmental protection, and the development of carbon-based new materials. It underscores an urgency for breakthroughs in fundamental research and its application. Some coal enterprises, with a myopic focus on immediate economic gains, resort to aggressive mining, inadvertently augmenting carbon emissions due to technical factors. Trade-centric provinces reliant on energy exports have vast innovation potential concerning technological effects, especially given the high intra-industry coordination in research and development (R&D) and innovation. Notably, the transfer of energy-saving and emission-reducing technologies has bolstered low-carbon innovation, particularly in the thermal power industry.

Yet, outliers exist. Provinces such as Jiangsu and Jiangxi exhibited negative technological effects, echoing findings by Cheng [19]. It suggests a considerable scope for technology upgrades within these provinces. A detailed analysis revealed that factors like final demand and economies of scale were the primary contributors to provincial carbon emissions, while industrial linkage, product structure, and technology effects curtailed them. However, the technological effect associated with metal smelting and rolling processing showcased a positive trajectory, indicating an avenue for technological enhancements in this sector—a contributing factor to its second-ranking position in embodied carbon transfer, right behind the electricity and heat production sector.

As elaborated earlier, the electricity and heat production sector stood out as the principal conduit for embodied carbon transfer, constituting a majority of China’s trade-driven embodied carbon transfer and an even larger slice of inter-provincial trade. Future endeavours in China’s environmental conservation and green transition will be intrinsically tied to the adoption of clean energy. With current technological offerings, electricity remains the pre-eminent clean energy alternative, albeit with the caveat of coal combustion being its frequent companion. Meeting the soaring electricity demands of China’s provinces and cities is an imminent challenge. Given that inter-provincial trade incorporates electricity transmission, our study underscores the analysis of determinants in this domain to carve pathways for carbon emission reductions. The emphasis is on bolstering the energy efficiency and low-carbon technology credentials of the industrial chain through innovative solutions that resonate with market needs, equipment upgrades for energy conservation, and the phasing out of obsolete production facilities. Concurrently, leveraging the national carbon emission trading market to unearth innovative avenues for a low-carbon trajectory is deemed essential.

**Fig 10:**
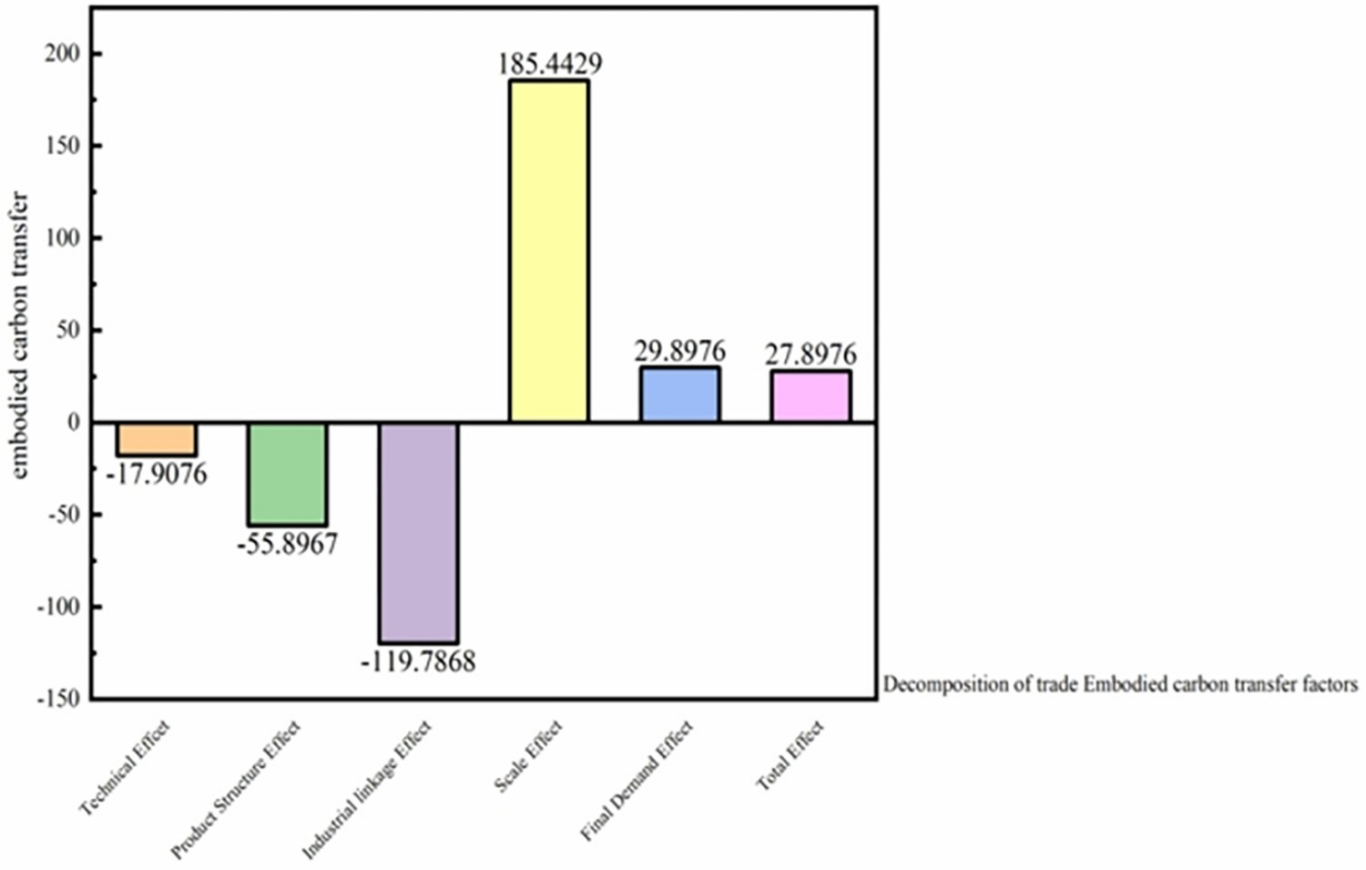
Decomposition of the embodied carbon structure in the production and supply of electricity and heat into the technology effect, product structure effect, scale effect, industrial linkage effect, and final demand effect (10 million tons). Note: Data derived from calculations. Refer to section 4.4 for the calculation method.

As evident from Fig 10, the decomposition of the embodied carbon structure within the production and supply of electricity and heat suggests that both the scale and final demand effects predominantly act as driving forces. Specifically, the scale effect augmented embodied carbon transfer by 1854.492 million tons, while the final demand effect contributed an increase of 298.976 million tons. Contrarily, the technological, product structure, and industry correlation effects served as restraining factors. These inhibiting effects reduced the embodied carbon transfer by 1197.868 million tons (industrial linkage), 558.967 million tons (industrial structure), and 1179.076 million tons (technical effects), respectively.

This analysis further highlights that embodied carbon transfer within China’s electricity and heat sectors represents a larger share in inter-provincial trade-driven emissions compared to those driven by final demand. Such a pattern is intrinsically tied to local economic developments. Presently, the primary source of thermal energy is electricity generation, which is heavily reliant on fossil fuels. Given the anticipated modest advancements in production technology in the foreseeable future, this can account for the subdued inhibitory role of technology within this sector. Nevertheless, China’s rigorous efforts to expand photovoltaic and wind power generation in recent years offer a promising pathway to mitigate fossil energy consumption. Such initiatives are poised to diminish the scale effect’s impact on the sector and amplify the adoption efficiency of clean energy solutions.

Recent works by Huang [37]and Su and Ang [38] emphasized the scale effect as a chief contributor, while the technological effect acts to diminish embodied carbon emissions. Nevertheless, it is still an open question whether the technological advantages of Chinese provinces are consistent. Our study delves into the nuanced roles of technological impacts across diverse Chinese regions, exploring the underpinnings of these patterns, thereby laying the groundwork for region-specific policy formulations."

## 6 Conclusions

For a robust economic system characterized by green, low-carbon, and circular development, understanding the pathways of inter-provincial carbon emission transfers is crucial to devise effective inter-regional carbon emission reduction strategies. While most studies emphasize embodied carbon emissions from international trade using Multi-Regional Input-Output model, this paper uniquely addresses China’s embodied carbon transfers within its industrial sectors and provinces in 2012, 2015 and 2017, providing more details about industry-specific dynamics. Drawing on the foundational principles of embodied flow theory, input-output theory, and the pollution paradise hypothesis, this study’s research hypotheses were rigorously examined. Using the multi-regional input-output model, we quantified the net carbon emission transfers grounded on provincial carbon emission inventories for the years 2012, 2015, and 2017. Beyond the traditional metrics of technology, industrial structure, scale, and final demand effects, we integrated the industrial linkage effect. This was to gain insights into the spatiotemporal dynamics of carbon transfers both between provinces and within industries, further validating the study’s hypotheses. Given China’s drive towards a green economy and the dual-circulation development model, understanding these domestic transfers becomes important for formulating suitable carbon reduction policies. The findings of this research are as follows:

1. China has witnessed a steady rise in its inter-provincial trade-embodied carbon transfers over recent years. The cumulative figures indicate that these transfers amounted to 4697.65 million tons in 2012, advanced to 5078.66 million tons by 2015, and further accelerated to 5256.86 million tons in 2017. Within a span of five years, this represents an increment of 559.21 million tons, equating to a growth rate of 11.90%. However, a nuanced time-series analysis suggests that certain provinces and industries within China have experienced a decline in their embodied carbon transfers.
2. While the primary sector has seen notable carbon transfers, it’s the secondary and tertiary sectors that predominantly drive such transfers. Upon close examination of the industries, it’s evident that the production and supply of electricity and heat emerge as the most significant contributors to carbon transfers. They are closely followed by metal smelting and rolling processing products, and non-metallic mineral products, ranking second and third, respectively. In 2012, inter-industry carbon transfers totalled 4697.65 million tons. Of this, the production and supply of electricity and heat were responsible for a substantial 50.15%, equating to 2355.86 million tons. Metal smelting and rolling processing products contributed 16.84% (or 791.13 million tons), while non-metallic mineral products represented 12.19%, amounting to 572.95 million tons. This hierarchy of leading carbon transfer industries—comprising the production and supply of electricity and heat, metal smelting and rolling products, and non-metallic mineral products—remained consistent in both 2015 and 2017.
3. Our analysis revealed that provinces with intensive energy consumption—specifically Hebei, Inner Mongolia, and Shanxi—dominated in total embodied carbon transfers from trade. Their respective transfers amounted to 437.8, 398.7, and 381.7 million tons in 2012; 367.8, 417.9, and 438.4 million tons in 2015; and 304.8, 298.7, and 289.2 million tons in 2017. Between 2012 and 2017, regions such as Fujian, Guangdong, Shanghai, and Xinjiang witnessed significant surges in embodied carbon transfers, registering growth rates of 114.21%, 101.24%, 85.43%, and 89.32%, respectively. Interestingly, provinces marked by rapid economic expansion exhibited reduced total carbon transfers. Furthermore, some provinces, despite having substantial embodied carbon transfers, experienced negative growth rates in this respect when juxtaposed with provinces registering lower transfers—Hebei and Shanxi being notable examples.
4. This research analysed China’s carbon transfer structure from 2012 to 2017, breaking it down into five determinants: technological advancements, product composition, industrial connections, growth scale, and end-user demand. Notably, the growth scale was the most dominant factor, escalating the inter-provincial trade’s embodied carbon transfer by 3,589.76 million tons. Conversely, end-user demand contributed to an increment of 489.76 million tons. The influence of industrial connections led to a reduction of 2,689.76 million tons. Technological effects and product composition effects further decreased the embodied carbon transfers by 478.96 million tons and 967.86 million tons, respectively.
5. In our research on the embodied carbon dynamics in electricity and heat production and supply, two factors were found to predominantly drive increases: scale and final demand, leading to embodied carbon transfer surges of 1,854.429 million tons and 298.976 million tons, respectively. In contrast, technological developments, product composition, and industrial connections displayed inhibitory effects. Specifically, the industry linkage, product structure, and technological impacts curbed the embodied carbon transfers by 1,197.868 million tons, 558.967 million tons, and 179.076 million tons, respectively.

## 7 Implications

### 7.1 Implications for research

1. As the embodied carbon transfers between provinces in China have grown significantly, especially within certain provinces and industries, it underscores the need for policymakers to prioritize these transfers in the pursuit of China’s "dual carbon" goals. Moreover, it emphasizes that at crucial developmental stages, we must seek emission reduction pathways that align with new development strategies and frameworks. This further suggests that, in order to adapt to the dual circulation industrial development model both domestically and abroad, we need to scientifically set benchmarks related to peak carbon and carbon neutrality. We hope that, within the broader context of global climate change, this will robustly drive the modernization process towards a harmonious coexistence between humans and nature.
2. Given the pivotal role of secondary and tertiary industries, especially in power production, metal smelting, and non-metallic mineral products, in implicit carbon transfers, tailor-made strategies for these sectors could substantially reduce emissions. As China’s inter-provincial carbon emissions shift from upstream to downstream in the industrial chain, regions in central and western China, such as power and heat production and supply, have emerged as the epicentre of high-carbon, resource-intensive industries leading the carbon emission spillover. Therefore, it’s imperative to deepen technological innovations aligned with market demands, advance energy-saving equipment transformations, and phase out outdated capacities, thereby enhancing the energy efficiency of the industrial chain and elevating low-carbon technology standards. Furthermore, we should fully leverage the advantages of the national carbon emissions trading market to explore new directions and strategies for low-carbon development.
3. The results illuminate the critical roles of Hebei, Jiangsu, and Henan in the arena of carbon transfers. Given their pronounced interactions with economically prosperous provinces and adjacent territories, there emerges a compelling prospect to nurture inter-provincial synergies. Such collaborations should champion carbon-responsible strategies, thoughtfully adapted to the distinct socio-economic and topographical nuances of each involved province.
4. By deconstructing the embodied carbon transfer structure into technology, product structure, industry correlation, scale, and final demand effects, this research elucidates the multifaceted determinants governing these transfers. As the scale dynamics tend to intensify, while the technological and product structural elements act to counterbalance the transfers, it becomes imperative for formulated strategies to holistically address these diverse underpinnings.

### 7.2 Implications for practice

1. Understanding the primary sectors and industries contributing to carbon transfers, practitioners can develop targeted interventions to mitigate carbon footprints, particularly focusing on secondary and tertiary sectors. In the pursuit of harmonizing economic development with environmental sustainability, attention must not only be given to the direct carbon emissions from major economic pillars like construction and service industries but also to their indirect carbon emissions. To achieve energy-saving and emission reduction objectives, it’s imperative to scientifically strengthen carbon emission standards in downstream industries across provinces and conduct in-depth analysis of their main trends in carbon transfer.
2. Recognizing energy-intensive provinces and their carbon transfers can help in crafting bespoke regional strategies, ensuring efficiency and effectiveness in tackling carbon emissions. The multi-faceted decomposition provides a clear roadmap for intervention. Focusing on influential factors like technology and product structure can have the dual benefit of advancing industries while reducing carbon transfers.
3. Economic growth and carbon efficiency can be harmonized, with growth trajectories guiding sustainable practices, transforming economic powerhouses into models of carbon efficiency. In defining carbon reduction responsibilities for each province, the spatial distribution characteristics of carbon emissions should be fully considered, acknowledging the implicit carbon transfers from developed to less-developed regions in interprovincial trade. By weighing each province’s carbon emissions from both producer and consumer perspectives, a rational distribution of carbon reduction responsibilities can be achieved.

### 7.3 Implications for society

1. Guided by the principles of Environmental Stewardship, understanding and managing inter-provincial carbon transfers advances a sustainable trajectory, allowing society to reap the rewards of economic growth without environmental detriment. Such comprehension, rich in its detail about key industries and provinces, paves the way for Informed Policy-making, enabling the crafting of policies that synergize both societal welfare and ecological responsibility.
2. Recognizing the dominant industries in carbon transfers accentuates the need for the Promotion of Sustainable Industries, urging society to champion more eco-friendly practices and innovations within these sectors. Simultaneously, the findings pave the way for enriched Regional Collaborations, enabling provinces to harness mutual learnings, capitalize on collective strengths, and address shared vulnerabilities, crafting a unified strategy for a greener future.
3. The analysis of China’s carbon transfer structure offers a lucid understanding of the underlying drivers contributing to the surge in carbon transfers. Armed with this insight, individuals, communities, and industry sectors can strategize and implement informed interventions that tackle the core issues, be it through technological advancements, refining product frameworks, or cultivating industry collaborations that emphasize ecological well-being.

Although this study makes significant contributions to the literature on China’s interprovincial embodied carbon emission space and industrial transfer path, the research content is not sufficiently comprehensive and needs further improvement. In the future, this study can be improved in two ways.

1. The effects of sector aggregation: this study integrates the 42 industrial sectors in various provinces and cities in the multi-regional input-output table for the years 2012, 2015, and 2017 into 28 research sectors. In the future, Su et al.’s [39] research can be used as a reference to analyse the possible embodiment of different industry clusters in the research results.
2. The effects of spatial aggregation: this study only analyses the embodied carbon transfer of 31 provinces in China and does not consider the different spatial aggregation levels. According to Su and Ang [39], China can be divided into eight.

## 8 Copyright statement

In this paper, the Chinese map was based on the Chinese government website http://bzdt.ch.mnr.gov.cn/browse.html?picId=%224o28b0625501ad13015501ad2bfc0692%22. In addition, the lines and colours were drawn by authors.

## Supporting information

S1 Table Summary of literature on MRIO analysis of China’s regional embodied flows. (DOCX)

S2 Table Overview of literature on SDA of China’s regional emission issues. (DOCX)
