# Supplementary material for: Inter-provincial embodied carbon emission space and industrial transfer paths in China
Source: PLoS One. 2024 Jun 27;19(6):e0300478. doi: 10.1371/journal.pone.0300478 (PMC11210824; doi:10.1371/journal.pone.0300478)
Supplement: S1 Table — (DOCX) [file pone.0300478.s001.docx]

**Table S1**. Summary of Literature on MRIO Analysis of China’s Regional Embodied Flows.

| Author | Year | Method | Advantages | Disadvantages |
| --- | --- | --- | --- | --- |
| Qiang Wang；  Xinyu Han | 2021 | the multi-regional input–output (MRIO) | This study innovatively uses the multi-regional Input-Output (MRIO) to analyze the concrete embodiment of carbon emissions in bilateral trade, providing a unique perspective for studying the impact of international trade on the environment. | While the research covers important aspects of carbon emissions embodied in trade, it primarily focuses on the Sino-US trade. Expanding the scope to include more countries or regions could provide a broader perspective. |
| Suocheng Dong;  Bing Xia;  Fujia Li;  Hao Cheng;  Zehong Li;  Yu Li;  Wenbiao Zhang;  Yang Yang;  Qian Liu;  Shantong Li | 2023 | the multi-regional input–output (MRIO) | Combined with cutting-edge research on global climate change, the goal of this study is to effectively guide the transition to low-carbon tourism by adopting new tools and strategies, providing valuable insights into green and low-carbon development in the post-pandemic era. | The study relies on extensive data sources and modelling, which may limit its applicability in regions or countries with less robust data infrastructure. |
| Zhencheng Xing;  Ziheng Jiao;  Haikun Wang | 2022 | Nested Multi-Regional Input-Output Table: | The study takes a comprehensive approach to understanding urban carbon emissions by analyzing how cities obtain, distribute, and manage their carbon emissions. This approach provides a more complete picture of the carbon footprint of urban agglomerations. | While the study mentions the recommendation of mitigation policy methods, it does not delve into the challenges and feasibility of implementing these policies in different types of cities. |
| Xiaoyu Li;  Zhao Zeng;  Zengkai Zhang;  Ye Yao;  Huibin Du | 2023 | Multiregional Input-Output (MRIO) Model; | The application of the MRIO model allows for a comprehensive assessment of embodied carbon emissions and their interplay within different regions. This modeling approach provides a robust foundation for the study's findings. | The research primarily concentrates on changes in consumption and export as driving forces behind carbon transfers. Broader factors, such as policy changes and economic development patterns, which may also influence these transfers, are not extensively explored. |
| Justin Caron;  Gilbert Metcalf;  John Reilly | 2014 | a mult-regional input-output (MRIO) framework | The study utilizes an MRIO model with a detailed, state-level breakdown and takes into account bilateral trade flows between US states and foreign countries. This improved data quality helps challenge critical assumptions and enhances the accuracy of emissions attribution. | The study is primarily focused on the United States, which may limit its generalizability to other countries and regions. The findings might not fully represent global patterns, and additional research in different contexts is needed to make broader comparisons. |
