# Supplementary material for: Inter-provincial embodied carbon emission space and industrial transfer paths in China
Source: PLoS One. 2024 Jun 27;19(6):e0300478. doi: 10.1371/journal.pone.0300478 (PMC11210824; doi:10.1371/journal.pone.0300478)
Supplement: S2 Table — (DOCX) [file pone.0300478.s002.docx]

**Table S2**. Overview of Literature on SDA of China’s Regional Emission Issues.

| Author | Year | Method | Advantages | Disadvantages |
| --- | --- | --- | --- | --- |
| H. Wang；  B.W. Ang；  Bin Su | 2017 | Index decomposition analysis；  structural decomposition analysis | Built on the Input-Output (I-O) model, offering a detailed understanding of energy consumption and emissions from an economic systems viewpoint.  Strongly linked to the economic system, focusing on supply-demand linkages.  Provides insights into production technologies, demand-side effects, and trade-related issues.  Helps in understanding energy or emissions reductions along global/national supply chains. | The underlying differences in some core concepts and the meanings of the drivers of change between IDA and SDA may introduce complexities. |
| Wen-Hao Xu;  Yu-Lei Xie;  Ling Ji;  Yan-Peng Cai;  Zhi-Feng Yang;  De-Hong Xia | 2022 | environmentally-extended multi-regional input-output (EE-MRIO); weighted average structural decomposition analysis (WA-SDA) models | Utilizes environmentally-extended multi-regional input-output (EE-MRIO) and weighted average structural decomposition analysis (WA-SDA) models, ensuring a thorough and detailed study. The study not only quantifies CFs but also pinpoints the socio-economic factors driving these footprints, aiding targeted interventions. | There's a noticeable shift of CFs from affluent provinces to underdeveloped regions in western China, which may lead to more challenges in emission mitigation for those areas. The need to focus on changes in per capita consumption and population dynamics, especially in populous regions, means that fluctuations in these parameters can affect CF mitigation efforts. |
| Junna Yan;  Yingzhu Li;  Bin Su;  Tsan Sheng Ng | 2022 | the multi-region input-output model (MRIO); structural decomposition analysis (SDA) | The research uses the MRIO (multi-region input-output) framework, enabling a detailed investigation from both regional and demand perspectives. Changes in regional energy use are systematically examined using both additive and multiplicative SDA frameworks, offering multiple perspectives. | While the study covers 31 regions, including Tibet, the specific findings or unique energy challenges and contributions of each region aren't explored in depth. |
| Shi-Chun Xu;  Le Zhang;  Yuan-Tao Liu;  Wen-Wen Zhang;  Zheng-Xia He;  Ru-Yin Long;  Hong Chen | 2017 | the structural decomposition analysis | The study extended the existing SDA model to decompose the carbon emissions increment into more effects (nine effects). | While the study breaks down many effects, certain effects like the technology effect do not appear to have been deeply explored or prioritized. |
| Guangxin Cui;  Yadong Yu;  Li Zhou;  Hongyu Zhang | 2020 | the logarithmic mean Divisia index | The study highlights the role of imported green power in significantly reducing carbon emissions in Beijing. The results provide actionable insights, specifically emphasizing the energy intensity effect's role in emissions mitigation. | While the article mentions the driving forces behind carbon emissions and Beijing's efforts to combat them, it doesn't delve deeply into how other cities or countries are approaching similar challenges or how Beijing's approach compares to best practices globally. |
| Sha Peng;  Xiao Wang;  Qian Du;  Kerong Wu | 2022 | structural decomposition analysis (SDA) | Through structural decomposition analysis (SDA), the study pinpoints key driving factors and sectors affecting household carbon emissions. By comparing income-based and consumption-based results, the study identifies new critical sectors responsible for changes in household emissions. | The study focuses on the environmental aspects, but it doesn't discuss the broader socio-economic implications of its findings, especially considering China's rapid urbanization and changing economic landscape. |
| Ruichao Li;  Ying Wei;  Meng Yi;  Lulu Zhang;  Baodong Cheng;  Chang Yu | 2022 | structural decomposition analysis (SDA) | The study analyzes the spatiotemporal characteristics of embodied timber carbon stocks, allowing for a nuanced understanding of stock changes over time and across regions. | The study might overemphasize the role of certain countries (e.g., China and India) without adequately addressing the roles of other significant players in the BRI region. |
| Zhipeng Tang;  Haojie Yu;  Jialing Zou; | 2022 | a three-tier structural decomposition analysis (SDA) | The research uses a three-tier structural decomposition analysis (SDA), which is a unique methodological approach to evaluate the effects of production substitution on China's ECEE, enhancing the accuracy and depth of the findings. | Even though the three-tier SDA provides depth, relying on a single method might miss out on nuances that could be captured using a multi-method approach. |
| Heming Wang;  Xinyu Li;  Xin Tian;  Lin Ma;  Guoqiang Wang;  Xinzhe Wang;  Zhi Wang;  Jiashi Wang;  Qiang Yue | 2022 | the structural decomposition analysis (SDA) | By employing the structural decomposition analysis (SDA) method based on an input-output model, the study ensures accurate and data-driven insights into resource intensity changes. | While identifying the construction sector as the most resource-intensive might be accurate, the study may inadvertently overshadow the need to address other sectors with significant resource intensities. |
| Changjian Wang;  Fei Wang;  Xinlin Zhang;  Yang Wang | 2022 | the logarithmic mean Divisia index  ;the structural decomposition analysis (SDA) | The study provides a comprehensive evaluation of coal consumption in Guangdong, considering both supply and demand factors. The use of both the logarithmic mean Divisia index (LMDI) method and the structural decomposition analysis (SDA) method ensures a rigorous and in-depth analysis of the driving factors. | The study zeroes in on coal, potentially sidelining the role and implications of other energy sources in the region. |
| Xiaoyu Liu;  Lixiao Zhang;  Yan Hao;  Xuemei Yin;  Zhimin Shi | 2022 | structural decomposition analysis (SDA) | Unlike many previous studies that either looked at national aggregates or specific regions, this research delves into the provincial heterogeneity, giving a granular perspective. | By concentrating only on urban household ECEs, the study potentially neglects the dynamics and impacts of rural household ECEs. |
| He Huang;  Jingke Hong;  Xianzhu Wang;  Alice Chang-Richards;  Jingxiao Zhang;  Bei Qiao | 2022 | structural decomposition analysis (SDA);  a combination of spatial independent variable lag model (SLX) | The study employs a combination of spatial independent variable lag model (SLX) and structural decomposition analysis (SDA), ensuring a comprehensive understanding of energy interaction patterns. The province-level assessment helps in understanding regional intricacies, allowing for a deeper dive into energy consumption dynamics in China. | Given the focus on spatial and regional heterogeneity, there might be an overemphasis on regional patterns, potentially neglecting macro factors. |
